# Integrated single-base resolution maps of transcriptome, sRNAome and methylome of *Tomato yellow leaf curl virus* (TYLCV) in tomato

**DOI:** 10.1038/s41598-019-39239-6

**Published:** 2019-02-27

**Authors:** Álvaro Piedra-Aguilera, Chen Jiao, Ana P. Luna, Francisco Villanueva, Marc Dabad, Anna Esteve-Codina, Juan A. Díaz-Pendón, Zhangjun Fei, Eduardo R. Bejarano, Araceli G. Castillo

**Affiliations:** 1 Instituto de Hortofruticultura Subtropical y Mediterránea La Mayora (IHSM-UMA-CSIC), Área de Genética, Facultad de Ciencias, Universidad de Málaga, E-29071 Málaga, Spain; 2 Instituto de Hortofruticultura Subtropical y Mediterránea La Mayora (IHSM-UMA-CSIC), Plant Virology group, E. E. La Mayora CSIC, Algarrobo-Costa, E-29750 Málaga, Spain; 3 Boyce Thompson Institute for Plant Research, Cornell University, Ithaca, New York, USA; 4 CNAG-CRG, Barcelona Institute of Science and Technology (BIST), E-08028 Barcelona, Spain; 5 Universitat Pompeu Fabra (UPF), E-08003 Barcelona, Spain

## Abstract

Geminiviruses are plant ssDNA viruses that replicate through dsDNA intermediates and form minichromosomes which carry the same epigenetic marks as the host chromatin. During the infection, geminiviruses are targets of the post-transcriptional and transcriptional gene silencing machinery. To obtain insights into the connection between virus-derived small RNAs (vsRNAs), viral genome methylation and gene expression, we obtained the transcriptome, sRNAome and methylome from the geminivirus *Tomato yellow leaf curl virus*-infected tomato plants. The results showed accumulation of transcripts just at the viral ORFs, while vsRNAs spanned the entire genome, showing a prevalent accumulation at regions where the viral ORFs overlapped. The viral genome was not homogenously methylated showing two highly methylated regions located in the C1 ORF and around the intergenic region (IR). The compilation of those results showed a partial correlation between vsRNA accumulation, gene expression and DNA methylation. We could distinguish different epigenetic scenarios along the viral genome, suggesting that in addition to its function as a plant defence mechanism, DNA methylation could have a role in viral gene regulation. To our knowledge, this is the first report that shows integrative single-nucleotide maps of DNA methylation, vsRNA accumulation and gene expression from a plant virus.

## Introduction

Geminiviruses constitute a large family of plant viruses with circular single-stranded DNA (ssDNA) genomes packaged within geminate particles^1,2^. In the *Geminiviridae* family, *Begomovirus* is the largest genus and comprises most of the viral species infecting dicotyledonous plants. Geminiviruses amplify their single-stranded genomes in the nuclei of infected cells by three replication strategies: complementary strand replication (CSR), rolling circle replication (RCR) and recombination-dependent replication (RDR)^3,4^. RCR and RDR are responsible for the accumulation of geminiviral DNA in multiple forms during infection: circular ssDNA, which is the molecule found in viral particles, circular dsDNA replicative forms, that are the templates for replication and transcription, and linear forms, some of which may be products of recombination-dependent replication^5^.

Tomato yellow leaf curl disease (TYLCD) is one of the most devastating viral diseases affecting tomato crops and it is caused by a complex of phylogenetically related Begomovirus ssp^6,7^. *Tomato yellow leaf curl virus* (TYLCV), the first known causal agent of TYLCD^8^, is a monopartite begomovirus which is transmitted by the whitefly *Bemisia tabaci*. The genome of this virus is bi-directionally organized in two transcriptional units (Left, L and Right, R) separated by an intergenic region (IR)^9,10^. The virion-sense strand (VS strand) contains two open reading frames (ORFs) in the R unit, named V2 and V1, that encode V2 and CP proteins, respectively. The complementary-sense strand (CS strand) encompasses four ORFs, C1, C2, C3 and C4, that are expressed from the L unit and that encode the proteins, Rep, TrAP, REn and C4, respectively. The role of the proteins encoded by these ORFs has been extensively reviewed elsewhere^5,9^. The IR encompasses the origin of replication, which includes a stem-loop structure containing a conserved nonanucleotide sequence required for the cleavage and joining of the viral DNA during replication^11^ and the regulatory DNA replication elements needed for Rep binding (iterons)^12,13^. Besides, it also includes the promoters and their regulatory sequences for transcription of all the ORFs: two TATA boxes located at either side of the stem loop and several cis-regulatory elements (CREs)^10,14,15^. Additional promoters and regulatory elements have been described for some geminiviruses, including one located in the C1 ORF that drives the expression of transcripts for the C2 and C3 ORFs^15^.

RNA silencing is a ubiquitous eukaryotic gene regulation mechanism and an important antiviral defence mechanism in plants^16^. Briefly, viral double-stranded RNA (dsRNA) which may derive directly from convergent transcription, transcription of inverted-repeat sequences or sequences with internal stem-loop structures, is recognized by a set of Dicer-like (DCL) ribonucleases and processed into 21 to 24-nt viral small RNAs (vsRNAs). VsRNAs associate with distinct Argonaute (AGO)-containing effector complexes which provide targeting specificity for RNA dicing (post-transcriptional gene silencing, PTGS) or chromatin modification, i.e. DNA methylation and histone modification (transcriptional gene silencing, TGS). Several reports have shown that there are functional homologues of the *Arabidopsis* genes involved in the RNA silencing pathway in tomato^17,18^. For a comprehensive picture of the silencing pathways there are excellent reviews^16,19,20^. DNA methylation is an important epigenetic mark that regulates gene expression and silencing of transposable elements and repeats^21^. RNA-directed DNA methylation(RdDM) is a regulatory pathway in which 24-nt siRNAs target *de novo* methylation of cytosines in all classes of sequence contexts, that is, CG, CHG and CHH (where H represents A, T or C). Plant’s RdDM depends on a specialized transcriptional machinery, POL IV and POLV, whose action recruits *de novo* methyltransferases DOMAINS REARRANGED METHYLTRANSFERASE 1 and 2 (DRM1 and DRM2), which leads to cytosine methylation in the three contexts^22^. A complex set of maintenance mechanisms, that involve maintenance DNA methyltransferases and histone modifying enzymes, ensure the persistence of established DNA methylation^21,23,24^.

Geminiviruses must confront PTGS and TGS^25–28^. Several works have deep-sequenced vsRNA populations from different geminivirus-infected plants such as tomato^29–32^, *Nicotiana benthamiana*^30^, *Arabidopsis thaliana*^33^ or cassava^34^. Although the results slightly vary depending on the geminivirus-host assayed, generally 21, 22 and 24-nt vsRNAs are the most abundant size classes. Geminiviral sRNAs are produced from both viral strands, cover almost the entire circular viral genome and accumulate at elevated levels in several hotspot regions. There are two reports on TYLCV vsRNAs generated during the infection in tomato which provide limited information, since the data came from a reduced number of reads (1212 reads)^29^ or belonged to field-collected tomato samples infected in most cases, with other tomato viruses^32^.

Geminivirus double-stranded DNA (dsDNA) associates with host histones to form minichromosomes^27,35,36^ which carry the same epigenetic marks as the host chromatin, i.e. DNA methylation at cytosines and histone modifications^37–39^. The level of geminiviral DNA methylation in infected plants depends on the geminivirus-host analysed, ranging from 50% to 2%^37,39–49^. In general, low levels of DNA methylation are reported for geminiviruses, which has led to the hypothesis that geminiviruses evade repressive cytosine methylation and TGS very efficiently by their resourceful replication mechanisms^27,45^. On the other hand, in tissues showing host recovery, an increase of viral DNA methylation levels or minichromosome compaction is detected, supporting an active role of TGS in host defence^37,39,41,47,50,51^.

To obtain insights into the connection between virus-derived small RNAs (vsRNAs), viral genome methylation and gene expression we have generated the transcriptome, sRNAome and methylome from the geminivirus TYLCV-infected tomato plants. In the current study, using Illumina high-throughput sequencing technology, we have obtained the transcriptome (mRNA-Seq) and sRNAome (sRNA-Seq) of TYLCV at different time points during the infection, and compared two viral inoculation methods: the “artificial” but widely-used agroinoculation and the infection by its natural vector, the whitefly *B. tabaci*. Moreover, using bisulfite treatment and deep sequencing (BS-Seq), the viral methylome was analysed at a time point in which the infection was well stablished, providing a reliable landscape for TYLCV methylation from tomato plants. Using this approach, we have generated single-nucleotide resolution integrated transcriptomic and epigenetic maps for TYLCV. This work represents the deepest molecular characterization at a single-nucleotide resolution, so far depicted for a geminivirus.

## Results

### TYLCV accumulation and gene expression in tomato is similar regardless of the inoculation method

**Figure 1:**
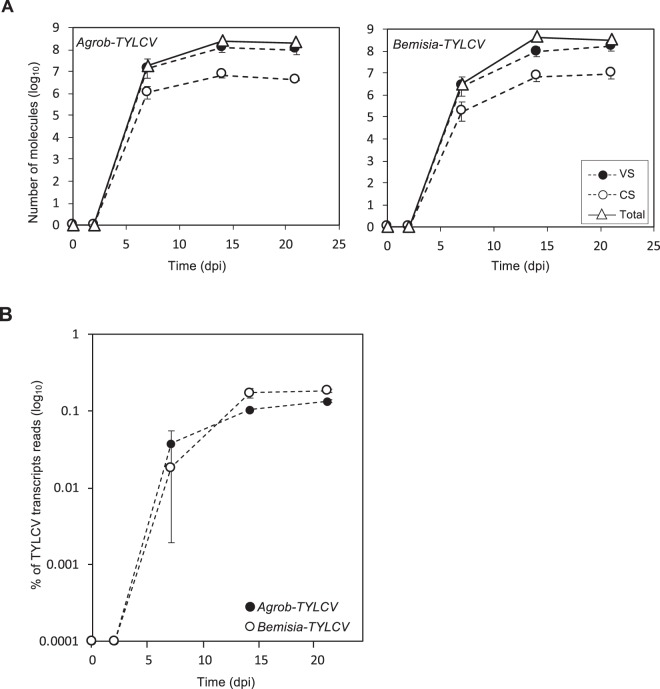
Genome and transcripts reads accumulation from TYLCV. Tomato plants were infected by either agroinoculation (*Agrob-TYLCV*) or using *B. tabaci* (*Bemisia-TYLCV*). Data from different time points during the systemic infection are shown (2, 7, 14 and 21 dpi). (**A**) Absolute quantification of virion-sense (VS) or complementary-sense (CS) strands and total viral DNA of TYLCV. Total DNA was analysed by two-step qPCR to quantify VS or CS strands. Total viral DNA was quantified by standard qPCR. Values represent the average ± SE of viral molecules per ng of DNA from the three biological replicates (log_10_). **(B)** Percentage of transcripts reads that mapped to TYLCV genome relative to total RNA-Seq transcripts reads. Data are represented by the mean ± SE. Values are the mean of the three biological replicates.

Three-week-old tomato plants were infected with TYLCV by agroinoculation or the whitefly *Bemisia tabaci (*referred from now on as *Bemisia*). Plants exposed to aviruliferous whiteflies or *Agrobacterium* carrying an empty binary plasmid were used as negative controls. TYLCV symptoms appeared at 7 and 10 days post-inoculation (dpi) in agroinfected and whitefly-infected plants, respectively. At 14 dpi, all inoculated plants showed TYLCV symptoms, although these were slightly more advanced in agroinfected plants, and at 21 dpi all plants displayed severe symptoms. Apical leaf tissue was sampled at 2, 7, 14 and 21 dpi and three independent samples at each time point were collected to generate three biological replicates. DNA was extracted to quantify the accumulation of total viral DNA and virion-sense (VS) and complementary-sense (CS) strands^52^. No viral DNA was detected in the mock plants from both inoculation methods. The pattern of viral DNA accumulation was similar in infected plants treated with *Agrobacterium* or *Bemisia*, with the total amount of viral DNA increasing exponentially before reaching a *plateau* at 14 dpi at a similar level; 2.8 × 10^8^ ± 1.1 × 10^8^ and 4.3 × 10^8^ ± 0.9 × 10^8^ molecules per ng of extracted DNA, respectively (Fig. 1A). In accordance with the symptoms development, at 7 dpi, whitefly-treated plants showed fewer viral molecules (5.4 × 10^6^ ± 3.9 × 10^6^ per ng) than plants infected by agroinfiltration (2.1 × 10^7^ ± 0.8 × 10^7^ per ng) (Fig. 1A). Consistent with previous reports for other geminiviruses^52^, VS strands accumulated to higher levels than CS strands (from 13 to 22 times) at all time-points during the infection (Fig. 1A, Table S1).

To further characterize the infection, the viral mRNA landscape was determined. Total RNA was extracted from the same samples used for viral quantification and subjected to RNA-Seq. On average, 30 million 75 bp paired-end reads per sample (range, 25–56 million) were obtained (Table S2). About 0.13% to 0.18% of the reads per sample were mapped to the TYLCV genome at 21 dpi (Fig. 1B, Table S3). The kinetics of TYLCV mRNA accumulation was in accordance with the amount of viral DNA previously detected. As expected, at 2 dpi almost no viral transcripts were found (Fig. 1B, Table S3) and a clear increase was observed in samples collected at 7 dpi from both *Agrobacterium* and whitefly-treated plants, representing 0.04 ± 0.017% and 0.02 ± 0.016% of the total reads, respectively. At 14 dpi, the number of viral transcripts raised approximately 3 times for *Agrobacterium*- and 9 times for *Bemisia*-treated plants, reaching a maximum level of 0.10 ± 0.005% and 0.17 ± 0.025% of the total reads, respectively. Those percentages were maintained at 21 dpi (Fig. 1B, Table S3). No differences in viral mRNA accumulation dynamics was observed when comparing samples from whitefly- and *Agrobacterium*-mediated TYLCV infection.

**Figure 2:**
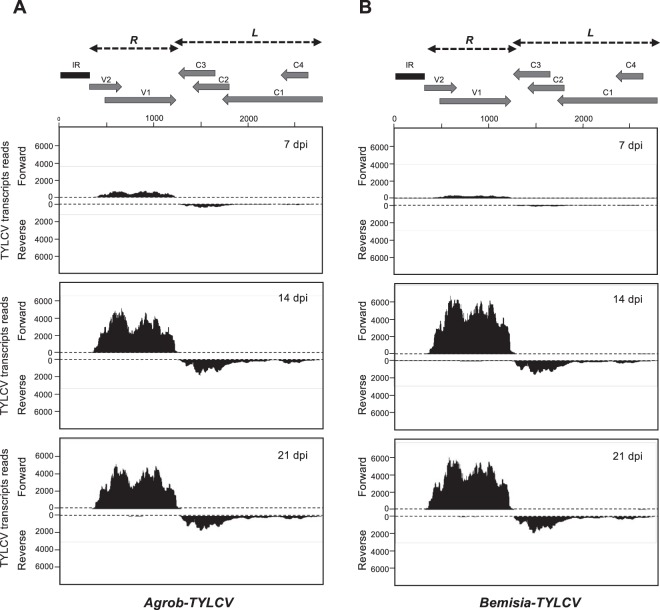
Maps of total transcript reads from TYLCV. Tomato plants were infected by either **(A)** agroinoculation (*Agrob-TYLCV*) or using **(B)** *B. tabaci* (*Bemisia-TYLCV*). The genome organization of TYLCV is shown schematically at the top. The predicted ORFs are shown as grey arrows and the IR as a dark rectangle. The R and L transcriptional units are indicated as black doted arrows. Data from different time points during the systemic infection are shown (7, 14 and 21 dpi). The graphs plot the number of viral transcripts reads on TYLCV genome (2781 nt). Bars above or below the axis represent sense (forward) or antisense (reverse) reads, respectively. One representative biological replicate is shown (replicate number is indicated in Table S2).

To determine the distribution of the viral transcripts accumulated during the infection, RNA-Seq reads were mapped against the viral genome. The TYLCV genome (2,781 nt presented in a linear form, setting the first nucleotide of the IR to 1 in Fig. 2) includes six ORFs distributed in two transcriptional units (R and L) separated by the intergenic region (IR) and transcribed from the VS or the CS strand, respectively. Our data indicated that viral mRNA reads were not homogeneously distributed along the genome and mapped according to the predicted ORFs with almost no reads mapping at the IR or at the antisense strands of the L and R transcriptional units (Fig. 2, Table 1). Transcripts from the VS strand accumulated to a higher level throughout the infection than transcripts from the CS strand in both infection methods. The reads in the VS strand (V1 and V2) represented around 75% of the total viral RNA-Seq reads at any time during the infection. Although transcription of both ORFs has been proposed to occur from the same promoter as a single transcriptional unit, the reads that mapped in the 5′ORF of V2 were less abundant than those corresponding to the V1 ORF (Fig. 2). The scenario was more complex for the four ORFs located at the CS strand (C1, C2, C3 and C4). The amount of reads for the C2 and C3 ORFs was significantly higher than the amount for the C1/C4 ORFs (Fig. 2). No significant differences in transcript accumulation levels of the different viral ORFs were detected among the samples collected at different times during the infection from plants treated with either *Bemisia* or *Agrobacterium*.

**Table 1:** Percentage of TYLCV transcripts reads that mapped to the virion-sense (VS) or complementary-sense (CS) strands.

*TYLCV transcripts reads per strand (%)*						
	*Agrob-TYLCV*			*Bemisia-TYLCV*		
	7 dpi	14 dpi	21 dpi	7 dpi	14 dpi	21 dpi
VS strand (R)	72.2 ± 21.4	74.7 ± 3.4	75.8 ± 4.7	72.5 ± 39.8	82.3 ± 7.9	80.0 ± 2.8
CS strand (L)	27.8 ± 6.0	25.3 ± 1.3	24.3 ± 0.7	27.5 ± 12.7	17.7 ± 1.1	20.0 ± 0.9

Tomato plants were infected by either agroinoculation (*Agrob-TYLCV*) or by *B. tabaci* (*Bemisia-TYLCV*). The average percentage (%) ± SE from the three biological replicates is shown at different time points during the systemic infection (7, 14 and 21 dpi). R and L viral transcriptional units are indicated.

#### 21, 22, and 24-nt TYLCV vsRNAs preferentially accumulate in tomato plants

**Figure 3:**
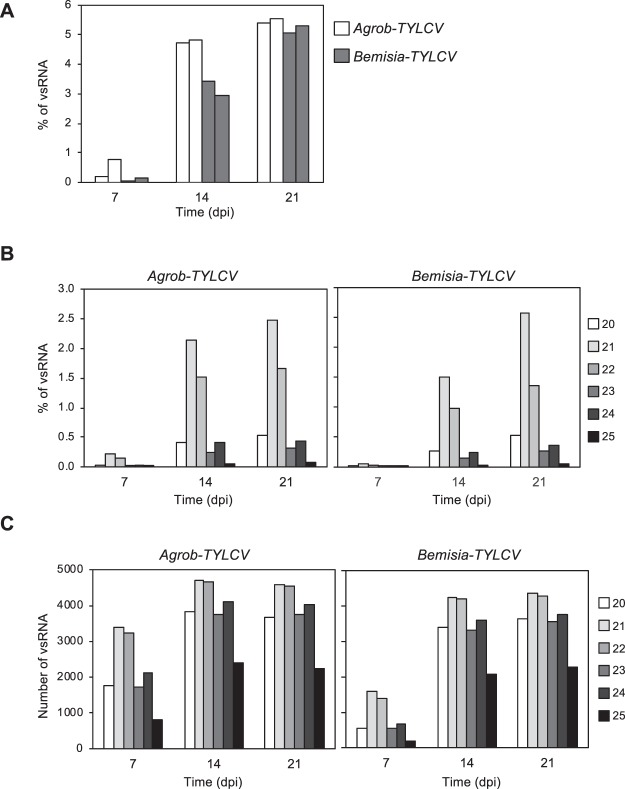
Accumulation and size distribution from TYLCV vsRNAs. Tomato plants were infected by either agroinoculation (*Agrob-TY*LCV) or by *B. tabaci* (*Bemisia-TYLCV*). Data from different time points during the systemic infection are shown (7, 14 and 21 dpi). **(A)** Percentage of vsRNA in the pool of 20–25 nt reads mapped to TYLCV genome relative to the total sRNA reads (19–35 nt). Each bar corresponds to an independent biological replicate. **(B)** Percentage of each size-class of 20–25 nt TYLCV vsRNA reads (redundant) relative to the total sRNA reads (19–35 nt). The bar from each vsRNA size, corresponds to the mean of the two independent biological replicates. **(C)** Total number of each size-class in the pool of 20–25 nt unique TYLCV vsRNA reads. The bar from each vsRNA size, corresponds to the mean of the two independent biological replicates.

Deep sequencing of sRNAs was performed on two of the three biological replicates from the TYLCV-infected plants used to determine the transcriptome at 7, 14 and 21 dpi (Table S2). Samples at 2 dpi were not analysed due to the low amount of virus at that time point (Fig. 1A, Table S1). The total number of raw sRNA reads ranged from 66 to 82 million (74 million on average, Table S2). As expected, the normalized amount of total (redundant) 20–25 nt sequences that mapped to TYLCV genome (vsRNA) increased along the infection, representing more than 5% of the total sRNA reads at 21 dpi (Fig. 3A). The kinetic of vsRNA accumulation was very similar to that observed for the viral DNA and mRNA. Although vsRNA were detected at 7 dpi in *Bemisia-* and *Agrobacterium*-treated plants, the levels of the latter were remarkably higher. The amount of vsRNAs strongly increased from 7 to 14 dpi (approximately 10 and 30 time-fold in *Agrobacterium* and whitefly-mediated TYLCV infection, respectively) and reached the highest accumulation at 21 dpi. No significant differences were detected between the two biological replicates of each infection method (Fig. 3A).

The level of redundancy of vsRNAs increased along the infection. At 7 dpi, low levels of redundancy were observed and the vast majority of the vsRNAs were read 1 to 10 times (around 77% in agroinfected plants and 89% in *Bemisia* TYLCV-infected plants, Table 2). As infection progressed, vsRNA that were read from 10 to 1000 times represented more than 42% of the total vsRNA, showing similar percentages at 14 and 21 dpi for plants infected by both methods (Table 2). The vsRNA size distribution of redundant vsRNA was similar to that previously reported in tomato plants infected with other monopartite begomovirus, such as *Tomato yellow leaf curl Sardinia virus* (TYLCSV), *Tomato yellow leaf curl China vi*r*us* (TYLCCNV) or TYLCV^30–32^. Our data showed that vsRNAs of 21 and 22-nt were the most abundant classes, representing approximately 3.5% of the total sRNA reads at 14 and 21 dpi (Fig. 3B, Table S4) and around 80% from the pool of 20–25 nt vsRNA reads at any time point studied (Table S5). vsRNAs of 20, 23 and 24-nt represented more than 15% of the 20–25 nt vsRNA population, being the 24-nt the most abundant among them (around 8%, Table S5). The size distribution pattern of the redundant vsRNA was maintained at all time points during the infection and no significant differences were detected between plants treated with the *Bemisia* or *Agrobacterium*.

**Table 2:** Redundancy of TYLCV vsRNAs.

Number of counts per vsRNA	*% vsRNA (20–25 nt)*					
	*Agrob-TYLCV*			*Bemisia-TYLCV*		
	7 dpi	14 dpi	21 dpi	7 dpi	14 dpi	21 dpi
1–10_VS_	37.35	27.31	26.97	42.04	28.35	26.47
1–10_CS_	40.04	27.41	27.58	47.21	29.05	26.64
10–100_VS_	9.25	16.12	15.93	4.63	15.76	16.42
10–100_CS_	10.10	17.13	16.77	5.26	16.33	17.42
100–1000_VS_	1.43	4.92	5.29	0.43	4.35	5.36
100–1000_CS_	1.66	5.57	5.74	0.41	4.96	5.81
>1000_VS_	0.09	0.70	0.79	0.01	0.57	0.89
>1000_CS_	0.08	0.85	0.93	0.01	0.63	1.00
**Total-Unique vsRNA**	13050	23424	22768	7366	20813	21813

Tomato plants were infected by either agroinoculation (*Agrob-TYLCV*) or using *B. tabaci* (*Bemisia-TYLCV*). Data from different time points during the systemic infection are shown (7, 14 and 21 dpi). Unique 20–25 nt vsRNA were classified according to the strand where they mapped (VS or CS) and to the number of counts in intervals 1 to 10, 10 to 100, 100 to 1000 and >1000 reads. The values indicate the percentage (%) of vsRNA according to the number of counts on each interval using the average from the two biological replicates. The total number of unique vsRNA (20–25 nt) at each dpi is shown.

The scenario was different for the unique vsRNA population (Fig. 3C). Although, 21 and 22-nt vsRNA were also the most abundant classes at all times, their relative proportion was reduced as the infection progressed. At 7 dpi, they represented 50 to 60% of all unique vsRNA, but this value decreased down to 40% in later times when the infection was well established (Table S5). The percentages for unique 20, 23 and 24-nt vsRNA were very similar between them and throughout the infection (Table S5). As 24-nt vsRNA is the third most represented size class of the redundant viral derived sRNAs at any time point (ranging from 6.9 to 8.8%), we can conclude that the redundancy of 24-nt vsRNA was higher compared to the 20-nt or the 23-nt classes (Table S5). Altogether our data indicated that most of the diversity of vsRNA already exists at 7 dpi. The significant increase of vsRNAs observed between 7 and 14 dpi was largely due to the production of additional copies of the same vsRNA, rather than the generation of new unique vsRNA. This redundancy increase was mainly caused by the rise in the total number of 21 and 22-nt size classes, although all the vsRNA sizes increased in a similar proportion during the infection (Tables S5 and S6).

### vsRNAs distribute along both strands of the entire viral genome and accumulate in several large hotspot regions

**Figure 4:**
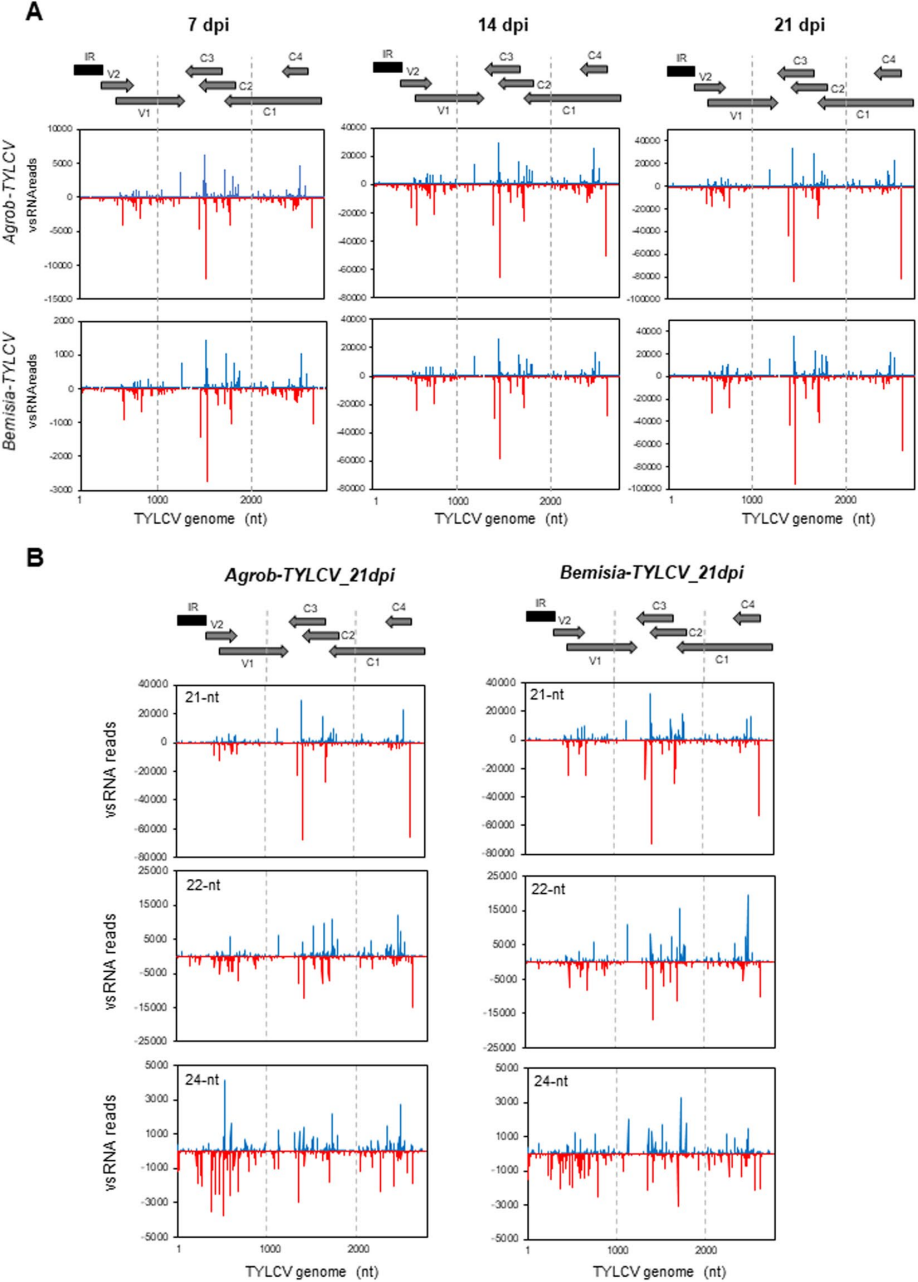
Maps from TYLCV vsRNA. Tomato plants by either agroinoculation (*Agrob-TYLCV*) or using *B. tabaci* (*Bemisia-TYLCV*). Data from different time points during the systemic infection (7, 14 and 21 dpi) are shown. The graphs plot the number of (**A**) 20–25 nt vsRNA or (B) 21, 22 and 24-nt vsRNA reads, at each nucleotide position of TYLCV genome (2781 nt). Bars above the axis (blue) represent sense reads starting at each respective position and those below (red) represent antisense reads ending at that position (please note the differences in the scale between 7 dpi and the other two time points). One representative biological replicate is shown (replicate number is indicated in Table S2). The genome organization of TYLCV is shown schematically above the graphs. The predicted viral ORFs are shown as grey arrows and the IR as a dark rectangle.

To examine the genomic distribution of the virus-derived sRNA, the reads from TYLCV-infected plants were plotted against the TYLCV genome. Analysis of single-nucleotide resolution maps of 20–25 nt vsRNAs revealed that they covered both CS and VS strands of the entire circular viral genome in an approximately equal ratio at 7, 14 and 21 dpi and regardless of the infection method used (Fig. 4A, Table S6), indicating that vsRNAs were generated from dsRNA precursors derived from both strands. Both sense and antisense vsRNAs displayed a heterogeneous distribution pattern along the genome with a large proportion of vsRNAs concentrating at specific areas, and this pattern was highly consistent between the two biological replicates at each time point assayed (Fig. S1). Three regions corresponding to the overlapping (OL) sequences between V2/V1, C1/C2/C3 and C1/C4, named here as OL1, OL2 and OL3 respectively, contained more than 60% of the vsRNAs and showed the highest density of vsRNAs per nucleotide (Table 3). Some of the highest accumulated vsRNAs on those hotspots were strand specific (Fig. 4A), suggesting the existence of potential strand-specific secondary structures used as DCL substrates. However, a good correlation between those vsRNAs and potential stem-loop structures predicted by “mfold Web Server” (http://unafold.rna.albany.edu/?q=mfold/rna-folding-form) could not be identified. VsRNAs distribution in tomato plants infected with two TYLCV-related begomovirus described the existence of similar vsRNA hotspots at OL1 (TYLCSV and TYLCCNV) and OL3 (TYLCSV) but not at the OL2 region^30,31^. The vsRNA hotspots on both strands were interspersed with sequences that engendered lower vsRNA abundance, including: (i) the regions leftward and rightward of the transcription start sites of the V2 and C4 ORFs respectively, that encompass the IR, accumulated the lowest levels of vsRNAs (Table 3, positions 2629–313); (ii) the region in which the R and L transcriptional units are expected to overlap and potentially form a dsRNA precursor and (iii) the region located at the 3′-end of the C1 ORF, in front of the predicted transcription start site of C2/C3 mRNA that could correspond to a putative promoter region in TYLCV (positions 1852–1824).

**Table 3:** Distribution of vsRNA abundance along TYLCV genome.


Region	Start	End	Size	*Number of vsRNA per nucleotide*						
				*Agrob-TYLCV (dpi)*			*Bemisia-TYLCV (dpi)*			
				7	14	21	7	14	21	
*IR*	1	313	313	30	207	232	6	114	192	
*V2*	314	472	159	76	739	615	5	413	543	
*V2/V1*	473	673	201	**343**	**1356**	**1336**	**72**	**1059**	**1430**	**OL1**
*V1-C3*	674	1391	718	119	569	481	75	476	587	
*C1/C2/C3*	1392	1799	408	**303**	**1627**	**1708**	**67**	**1317**	**2155**	**OL2**
*C1- 3′end*	1800	2336	537	109	534	488	23	361	473	
*C1/C4*	2337	2628	292	**255**	**1628**	**1741**	**55**	**1062**	**1616**	**OL3**
*C1- 5′end*	2629	2781	150	19	128	119	4	78	126	
*Genome*	1	2781	2781	146	832	818	31	616	893	

Tomato plants were infected by either agroinoculation (*Agrob-TYLCV*) or by *B. tabaci* (*Bemisia-TYLCV*) and data from different time points during the systemic infection are shown (7, 14 and 21 dpi). The values represent the number of vsRNA (20–25 nt) per nucleotide at distinct genomic regions according to the position of the viral 5′nucleotide. The regions are identified by the viral genes they encompass, their size (nt) and their coordinates according to the TYLCV genome map are indicated at the top. The three genomic regions that showed the maximum number of vsRNA per nucleotide (OL1, OL2 and OL3) are marked in bold. Data comes from the same biological replicates showed in Fig. 4A,B.

Most of the vsRNA hotspots contained all the three major size-classes of TYLCV vsRNAs, 21, 22 and 24-nt, suggesting that the same dsRNA precursors may be processed by different DCLs (Fig. 4B). However, a more careful analysis revealed that, while the distribution along the viral genome of 21 and 22-nt size classes was very similar, the 24-nt class showed a relative large accumulation at the IR at any time during the infection. The percentage of vsRNAs that were mapped to the IR was clearly higher for the 24-nt size class (from 7.4 to 9.1%) compared to the 21-nt (from 0.9 to 1.4%) or 22-nt (from 2.0 to 3.6%) classes (Table 4), indicating that the dsRNA substrate originated in that region is a preferential substrate for the DCL3 homologue which seems to be involved in the production of endogenous 24-nt sRNAs in tomato^18^. Interestingly, the majority of the 24-nt vsRNAs that accumulated at the IR were strand-specific, with 3 to 5-fold more 24-nt vsRNAs mapping to the CS than to the VS strand (Table 4).

**Table 4:** Percentage of 21, 22 and 24-nt vsRNAs classes at the IR from TYLCV.

*vsRNA at IR (%)*												
	*Agrob-TYLCV*						*Bemisia-TYLCV*					
	*21-nt*		*22-nt*		*24-nt*		*21-nt*		*22-nt*		*24-nt*	
	VS	CS	VS	CS	VS	CS	VS	CS	VS	CS	VS	CS
7 dpi	0.5	0.6	0.7	1.4	1.2	**6.4**	0.4	0.5	0.8	1.2	1.5	**7.5**
14 dpi	0.6	0.7	1.0	1.9	1.7	**5.7**	0.5	0.5	0.9	1.2	1.7	**5.5**
21 dpi	0.6	0.8	1.0	2.6	1.5	**7.6**	0.6	0.5	1.1	1.5	2.3	**5.7**

Tomato plants infected by either agroinoculation (*Agrob-TYLCV*) or by *B. tabaci* (*Bemisia-TYLCV*) and data from different time points during the systemic infection (7, 14 and 21 dpi) are shown. The percentage (%) represent the reads of each vsRNA size class from the IR (positions 1 to 313), at each strand (VS or CS) or from both strands (Total), relativized to the total TYLCV reads from that size. Data comes from the same biological replicates showed in Fig. 4A,B.

In summary, this vsRNA distribution pattern along the TYLCV genome indicated that during infection, viral dsRNA precursors present preferential internal excisions of vsRNA at certain regions or they produce vsRNA duplexes with differential stabilities. Moreover, the promoter and terminator regions on the TYLCV genome are depleted of highly abundant vsRNAs. No significant differences in the distribution of the vsRNA in the pool of 20–25 nt reads along the TYLCV genome, were observed among the samples collected at different times of the infection or between the plants treated by *Agrobacterium* or *Bemisia* (Fig. 4, Table 3, Fig. S1).

It has been reported that the 5′-terminal nucleotide of the sRNA directs its loading to different AGO complexes^53–55^. The nucleotide of the 5′-terminal position was analysed in the vsRNAs from *Agrobacterium* and *Bemisia*-treated plants. We found a preference of beginning with a uridine (U) or an adenosine (A) in a similar percentage (from 30 to 40% for each of the two nucleotides) and a clear tendency to avoiding guanidine (G) in vsRNAs from 20 to 25-nt regardless of the infection method and time point assayed (Fig. S2). In *Arabidopsis*, AGO2 and AGO4 preferentially recruit sRNAs with a 5′-terminal A, whereas AGO1 predominantly favours a 5′ terminal U^53–55^. Our results suggest that TYLCV vsRNA might be loaded into diverse AGO-containing silencing complexes in tomato, as previously reported for TYLCCNV^30^. This scenario is similar to the one described for CaLCuV (*Cabbage leaf curl virus*) vsRNAs generated in *Arabidopsis*-infected plants, which were also enriched in 5′A and 5′U (70 to 60%)^33^.

#### High resolution methylome of TYLCV reveals dense methylation levels at two distinctive regions

As previously mentioned, several reports have demonstrated that geminiviral DNA is methylated during infection and host recovery, and the level and extent of this methylation varies depending on the geminivirus and the host analysed. Nevertheless, they provide a slender picture of the geminiviral methylation pattern in infected plants, since the data is obtained by sequencing a limited number of RCA (Rolling Circle Amplification) or PCR-amplified clones, and in most cases, the level of methylation is determined only for a small region of the genome, generally the IR and flanking sequences. Here, deep sequencing of bisulfite-treated DNA (BS-Seq) was performed on the same two biological replicates of TYLCV-infected tomato plants used to determine the TYLCV transcriptome and vsRNA composition at 14 dpi. On average, 133 million 100 bp paired-end reads per sample (range, 128–144 million) were obtained (Table S2), which contained 65–107 K reads uniquely mapped to the TYLCV genome, representing an average TYLCV methylome of 5,000x and 7,500x in plants treated with *Agrobacterium* or *Bemisia*, respectively (Table S7). Thus, to our knowledge, this work presents the highest-resolution methylome of a geminivirus and provides a reliable landscape for TYLCV methylation in tomato plants.

**Figure 5:**
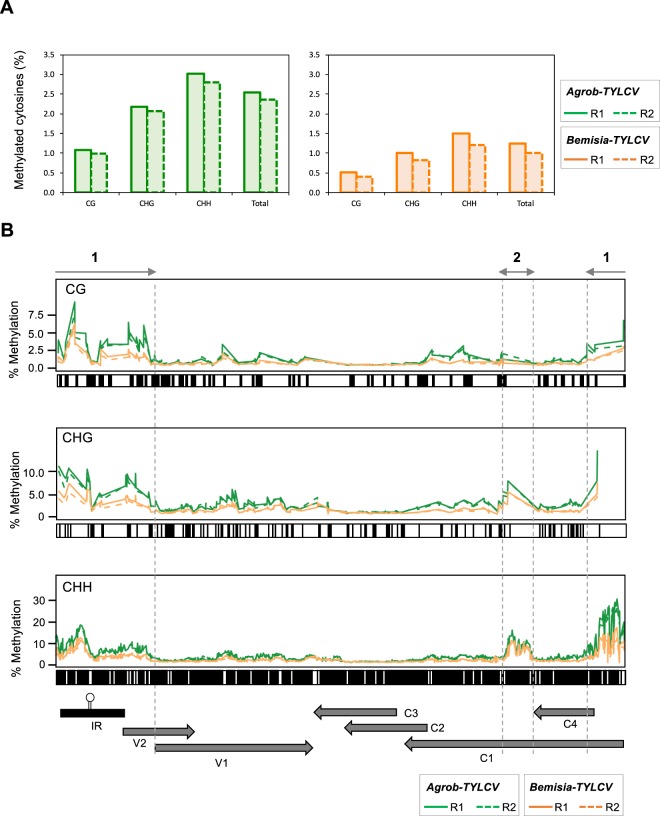
High resolution methyome of TYLCV. Tomato plants were infected by either agroinoculation (*Agrob-TYLCV*, green) or using the whitefly *B. tabaci* (*Bemisia-TYLCV*, orange). **(A**) The histogram shows the percentage of cytosine residues methylated at each cytosine context (CG, CHG and CHH) and the total methylation in TYLCV genome at 14 dpi. Each bar corresponds to an independent biological replicate (R1, solid line and R2, dotted line). **(B)** The graphs show the percentage of methylated cytosines in different contexts, CG, CHG and CHH, at each position in TYLCV genome (please note the differences in the scale between the three cytosine contexts). Each line corresponds to an independent biological replicate (R1, solid line and R2, dashed line). The position of each cytosine context on TYLCV genome, is represented by a dark line underneath each graph. The genome organization of TYLCV is shown schematically underneath the graphs. The two highest methylated regions are indicated by a grey solid line with arrowheads (1 and 2).

The percentage of methylated cytosines detected in TYLCV genome on each biological replicate ranged from 1.0% in *Bemisia*- to 2.5% in *Agrobacterium*-treated plants (Fig. 5A), which were similar to the ones found in the methylome of TYLCCNV infecting *N. benthamiana*^43^. The average TYLCV methylome coverage was greater in *Bemisia*- than in *Agrobacterium*-treated plants (Table S7), ruling out the possibility that the lower levels for TYLCV methylation detected in the whitefly-treated plants could be due to technical problems. A possible explanation for the differences detected in TYLCV DNA methylation between both methods could be the slight delay in the infection development observed at 7 dpi in the plants TYLCV-infected by the whitefly (Figs 1, 3, Tables S3 and S6). In spite of this difference, the relative proportion of methylated cytosines at the three contexts was similar by both infection methods, being the percentage of methylation at CHH and CG sites the highest and the lowest, respectively (Fig. 5A, Table S8).

Since the main target of geminiviral DNA methylation is dsDNA^45,48^, this apparently low level of methylation has to be taken cautiously. A more precise value of the percentage of methylated geminiviral DNA could be estimated considering the percentage of cytosines that form part of the viral dsDNA molecules. At 14 dpi, an average of 11% and 18% of the total viral DNA strands form dsDNA molecules in *Agrobacterium* and *Bemisia*-treated plants, respectively (Table S8). Taking into account this rough calculation, we could estimate that the percentage of methylated cytosines in viral dsDNA is around 23% for the agroinfiltrated samples and 7% for the  *Bemisia*-treated ones (Table S8). Moreover, a methylation value in a defined cytosine residue above 11% and 18% will indicate that, this residue will be methylated in all dsDNA viral molecules.

**Figure 6:**
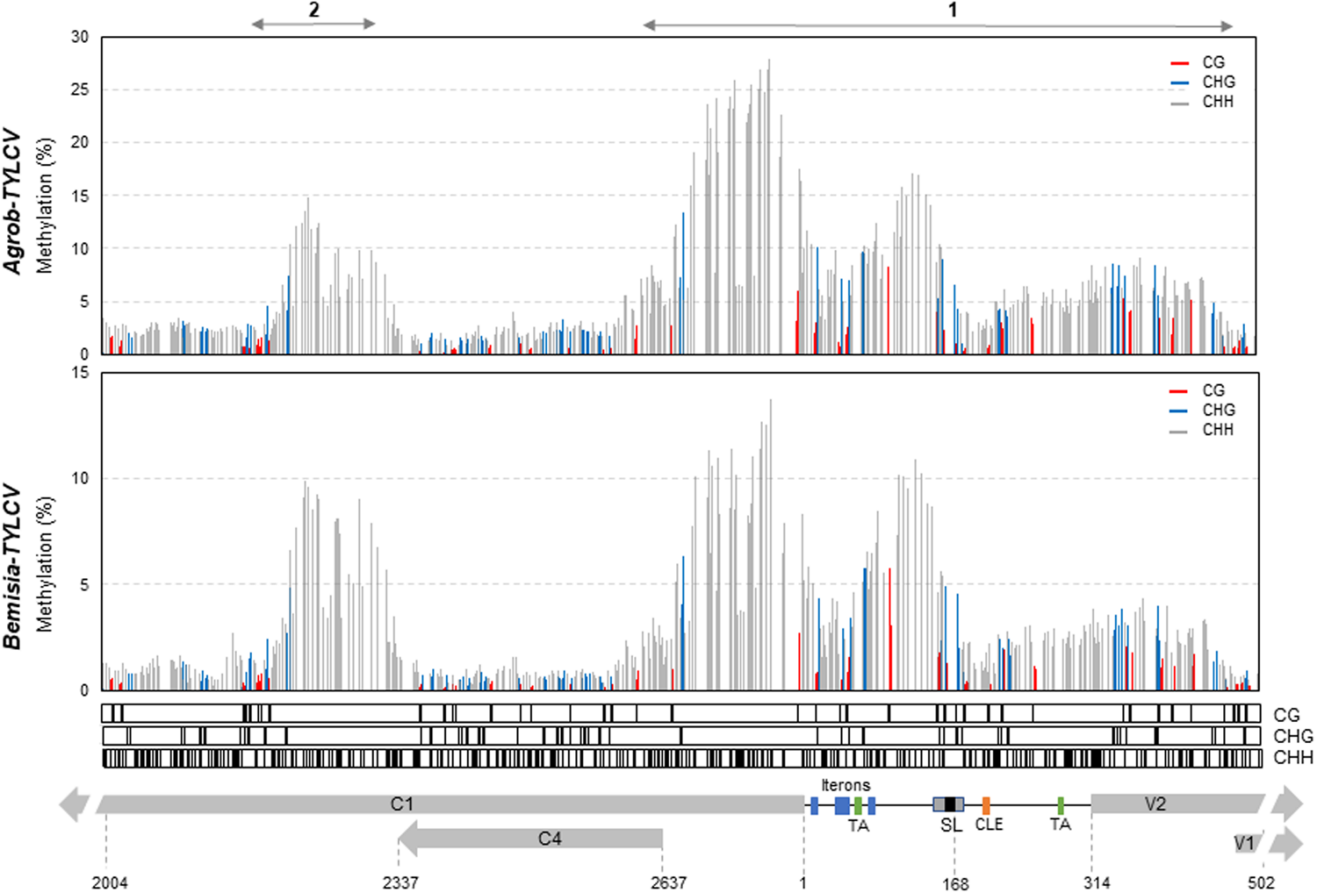
Detailed representation of TYLCV DNA methylation at the IR and flanking sequences. Tomato plants were infected by either agroinoculation (*Agrob-TYLCV*) or using the whitefly *B. tabaci* (*Bemisia-TYLCV*). The data correspond to the average of the two biological replicates shown in Fig. 5. Bars represent the percentage of methylation on each cytosine context (CG in red, CHG, in blue and CHH in grey). The position of each cytosine context on TYLCV genome, is represented by a dark line underneath the graph. TYLCV genome organization at the IR and surrounding sequences is indicated at the bottom and the regulatory elements from the IR are shown (SL, stem loop; Iterons of the Rep-binding region; TA, TATA box; CLE, conserved late element). The predicted viral ORFs are shown as grey arrows and relevant nucleotide positions are indicated. The two highest methylated regions are indicated by a grey solid line with arrowheads (1 and 2).

Interestingly, when checking cytosine methylation profiles across the viral genome, two distinctive regions with dense levels of cytosine methylation (marked as region 1 and 2) could be noticed in both infection methods, being highly consistent between the biological replicates (Fig. 5B). As previously described for other geminiviruses, we detected high levels of methylation at the IR that spanned rightwards and leftwards and drastically dropped at the beginning of the V1 and C4 ORFs (region 1). Nevertheless, this high-resolution approach let us dissect the methylation pattern at this region, characterized by containing two divergent promoters. The lowest level of methylation at the IR was located at the 3′-end of the stem loop, in a sequence that contains the putative C2 protein binding domain (positions 195 to 203; CLE, conserved late element)^14^ (Fig. 6). At the 3′-side of the stem loop, in the region that comprises the TATA box of V2/V1 transcripts and the 5′-end of the V2 ORF, a moderate increase in DNA methylation was observed. This methylation dropped dramatically just a few nucleotides before the start site of V1 (Fig. 6, region 1). At the 5′-side of the stem loop up to the C4-ORF start site, a larger rise in methylation levels was detected. This region, partially depleted of cytosines in the CG and CHG context, showed the highest level of DNA methylation in the viral genome (Fig. 5B). Interestingly, a slight reduction in the methylation level of this region was observed before the start site of the C1 ORF, in a sequence that contains the iterons of the Rep binging domain and the TATA box of the L transcriptional unit (Fig. 6). A second region (region 2), that spans approximately 150 nucleotides just downstream of the C4 ORF stop codon and that is also depleted from cytosines in CG or CHG context, contained elevated levels of cytosine methylation (positions 2182–2334) (Fig. 6). This methylation peak seems to be upstream of the region that encompasses the formerly described elements that act as an internal promoter for C2 and C3 genes in different geminiviruses^15,56,57^ and the nucleosome-free gap described for *Abutilon mosaic virus* (AbMV) transcriptionally active minichromosomes^48^.

## Discussion

Since the first report in 1988^58^, agroinoculation has been widely used and accepted as a standard method to infect plant with gemiviruses. Although, along these years the scientific community has assumed that the dynamics of geminiviral infections produced by agroinoculation, or by its natural vector, the whitefly *B. tabaci*, were similar, this is the first time that a thorough molecular analysis of a side by side infection using both inoculation methods has been carried out. The data showed no significant differences in either of the parameters analysed (accumulation of viral DNA, viral transcripts, vsRNA and DNA methylation pattern) among the samples collected from plants infected by the two methods. This result confirms that both are equivalent methods to study geminiviral infections.

**Figure 7:**
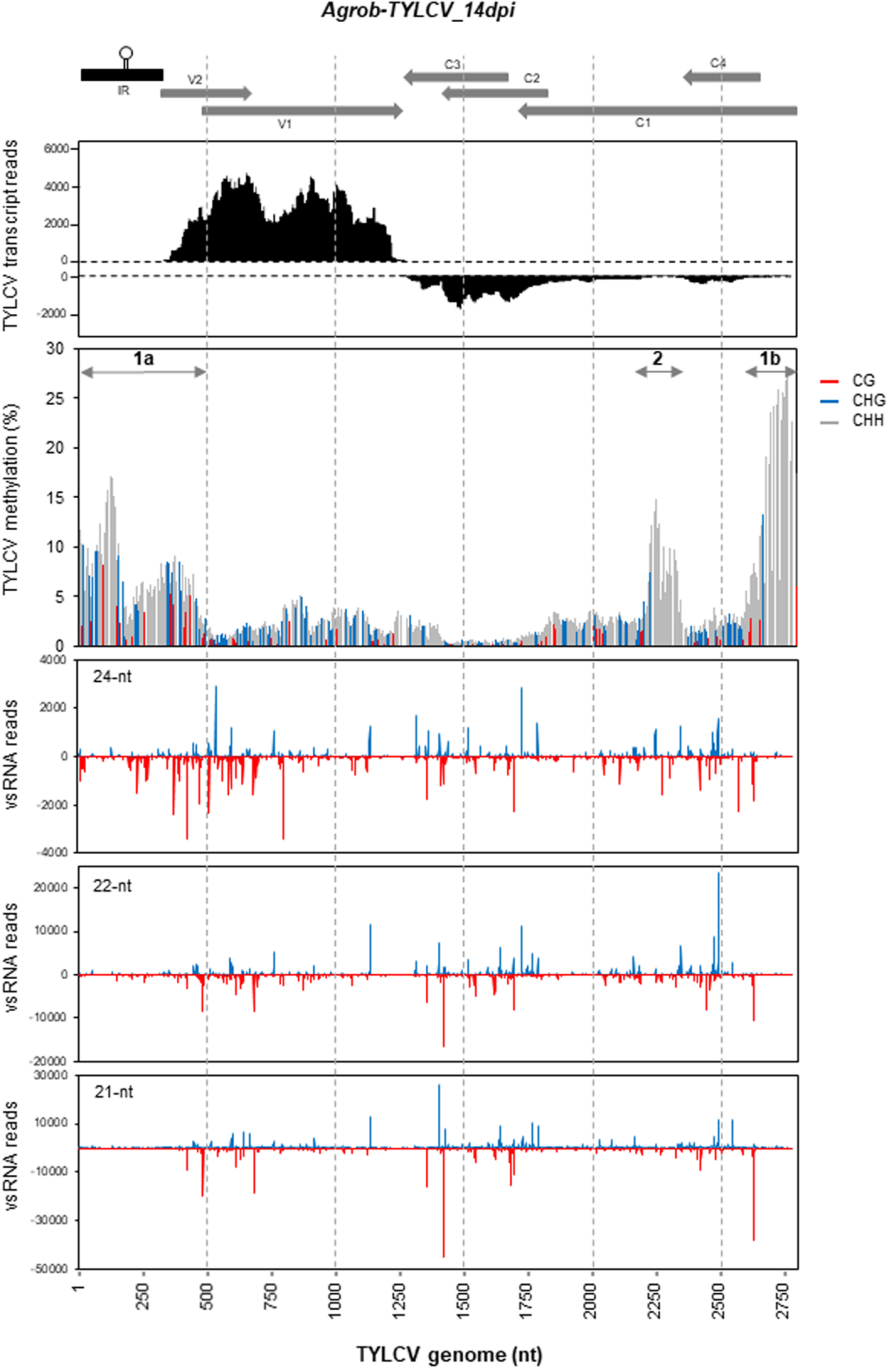
Maps of viral transcripts reads, DNA methylation and 24, 21 and 22-nt vsRNA reads, at each nucleotide position of TYLCV genome (2781 nt). Data from agroinfiltrated tomato plants (*Agrob-TYLCV*) at 14 dpi are shown. The genome organization of TYLCV is shown schematically above the graphs. Figure details are explained in Figs 2, 4B and 5B.

24-nts long siRNAs, along with Pol IV, Pol V, RDR2, DCL3 and AGO4, are the essential players of the canonical RdDM pathway, but several RdDM mechanisms which partly incorporate components associated with PTGS such as 21 and 22-nt siRNAs, namely non-canonical, have also been reported^22^. The fact that elements of the RdDM canonical pathway seem to be involved in geminiviral genome methylation^37,47,50^, suggests that vsRNAs could be directing viral DNA methylation. To better appreciate the connection between TYLCV vsRNAs distribution, viral methylome and transcripts levels, data from TYLCV-agroinfiltrated plants at 14 dpi were compiled in Fig. 7. We could detect high methylation at sequences containing or adjacent to the three geminiviral promoters but, in general, the promoter and terminator regions were depleted of highly abundant vsRNAs. The low representation of vsRNAs that we have found at the IR of TYLCV is in accordance with the results previously obtained by high resolution RNA blots^59,60^ or deep sequencing^30,31,33,34^ for other geminiviruses. Broadly, we did not find a clear correlation between 24, 21 and 22-nt vsRNA hotspots and viral DNA methylation peaks, as we could detect regions that contained a high density of 24-nt vsRNA and just residual cytosine methylation (Fig. 7, positions 1390 to1811) as well as other areas that contained high levels of DNA methylation and low amounts of 24, 21 and 22-nt vsRNA (Fig. 7, positions 2632 to 2871, 5′-end of C1). A detailed analysis of vsRNA distribution and DNA methylation at the promoters, described three different epigenetic scenarios. The region that contains the promoter for the R transcriptional unit showed elevated methylation levels at the three cytosine contexts and high density of 24-nts vsRNAs with limited presence of 21 and 22-nt vsRNAs, suggesting that cytosine methylation at this promoter region may be targeted by the canonical RdDM pathway (Fig. 7, region 1a). This pattern was maintained through the V2 ORF up to the start site of V1, where we could detect an abrupt drop in DNA methylation and an increase in the accumulation of 21 and 22-nt vsRNAs, while the density of 24-nt size class was maintained (Fig. 7, region 1a). A similar situation was observed at the L transcriptional unit promoter, with high levels of methylation and relative abundance of 24-nt vsRNAs, but with a low amount of the 21 and 22-nt classes (Fig. 7, region 1a). DNA methylation also extended along the 5′end of the C1 ORF (position 2632 to 2781) dropping sharply at the beginning of C4 ORF. However, the epigenetic profile for the 5′end of the C1 ORF was different from the V2 one, as it also showed high levels of DNA methylation (the highest on TYLCV methylome) but accumulated almost no vsRNAs of any size, suggesting that DNA methylation at this region was RdDM-independent (Fig. 7, region 1b). In plants, DNA methylation also occurs in a sRNA-independent manner and relies on chromatin features^61,62^, opening up the possibility that histone modifications in the viral minichromosome at that region renders the DNA methylation at the 5′-end of the C1 ORF. Surprisingly, the chromatin in that region from another begomovirus does not contain H3K9me2^38^, an epigenetic histone mark that could drive DNA methylation at the CHH context, opening the question about how DNA methylation is established in that region. We could detect a third epigenetic profile that corresponded to the methylation peak located around the middle of the C1 gene, just downstream the C4 ORF, which contained high levels of methylation, a high density of 24-nts vsRNAs from both strands and also a moderate amount of 21 and 22-nts vsRNAs (Fig. 7, region 2). This observation suggested that canonical and non-canonical RdDM mechanisms could be responsible for establishing and maintaining the methylation at this enriched CHH region. Interestingly, moderate but dense levels of methylation were observed in almost all cytosines upstream the region that encompasses the formerly described elements that act as an internal promoter for C2 and C3 genes in different geminiviruses, which is activated by the Rep-mediated repression of its upstream promoter located at the IR^15,56,57^. The higher accumulation of C2 and C3 transcripts reads from our transcriptomic data compared to C1 and C4 ones (Figs 2 and 7), supports the existence of an internal C2/C3 promoter also in TYLCV. The identification of the *cis* elements and the role that methylation could play in controlling its transcription, will need to be further characterized.

The type and amount of DNA methylation and its location within a gene (promoter or ORF) determines the impact of this epigenetic mark on its transcription. Our results indicated that in the viral population, both bidirectional promoters at the IR are methylated to a different extent, which could explain the differences found in the expression of the R and the L transcriptional units (Fig. 7, Table 1). As we previously mentioned, it is accepted that V2 and V1 genes are expressed from the same transcriptional R unit, but our data showed that the RNA-Seq reads from both ORFs accumulated to different levels (Fig. 2). The fact that a large region of the 5′-end of the V2 ORF (around 150 nt) showed high and dense DNA methylation levels (Figs 6 and 7), brings up the question whether that epigenetic mark could be involved in the lesser accumulation of V2 transcripts and favouring another transcription start site for V1. Interestingly, there is a TATA box (positions 448–451) 22-nt upstream of the V1 start codon (position 474) raising the possibility of an additional transcription start site that could produce specific transcripts for TYLCV V1. The same rationale could be applied to the C1 and C4 ORFs which share the same promoter at the IR. There is a minor accumulation of C1 transcripts compared to C4 in combination with high and dense levels of DNA methylation at its 5′-end just prior the start site of the C4 ORF.

The complex picture for the vsRNA profile and the viral DNA methylation landscape described here, poses the question whether the role of DNA methylation could be just narrowed down to a plant defence mechanism that transcriptionally silences the virus, or if the virus needs this epigenetic mark, and others, to properly regulate its gene expression. The mechanisms that lead to these distinct methylation/vsRNA accumulation patterns at the viral genome and the biological relevance of these epigenetic marks, will need further study and raise new exciting questions to the field.

## Materials and Methods

### Plant material and virus infection

Tomato plants (*Solanum lycopersicum* cv. Moneymaker) were grown in an insect-free growth chamber with a 16-h photoperiod at 250 μmol·s^−1^m^−2^ photosynthetically-active radiation (25 °C/20 °C) and 70% relative humidity. Three-week-old seedlings were infected with TYLCV (Genbank AC: AJ489258), either using *Agrobacterium tumefaciens* LBA4404 or *Bemisia tabaci*. A virus-free colony of *B. tabaci* MED species (former Q biotype), was reared on melon plants plants in cages covered by insect-proof netting. To obtain viruliferous whiteflies, non-viruliferous whiteflies were given a 24-h acquisition access period (AAP) on leaves of tomato plants infected with TYLCV by agroinoculation. Similarly, whiteflies used as non-viruliferous controls were enclosed in clip-cages attached to virus-free plants for the same 24-h period. After the AAP, whiteflies were transferred to healthy plants for a 48-h inoculation access period (40 whiteflies per test plant in clip-on cages). Agroinfection was performed as described in^63^.

### Sample collection and nucleic acid extraction and geminivirus quantification

TYLCV infected and control plants were monitored along 21 days. The second most recently expanded leaf from the apex was harvested at 2, 7, 14 and 21 days-post infection (dpi). The presence of viral DNA was analysed in each test plant at 21 dpi by hybridization of tissue blots^64^. For each infection method, time point and replica, the leaf tissue from 6 infected plants was pooled and used in downstream analysis. A total of three biological replicates were processed per condition and time point.

Total DNA was isolated with the DNeasy Plant Mini Kit (Qiagen). Total RNA was isolated using the Trizol reagent (Ambion), and 3 μg were set aside for construction and sequencing of small RNAs libraries. Total RNA was treated with RNase free Turbo DNaseI (Ambion) according to the manufacturer’s guidelines and cleaned up by a phenol:chloroform treatment.

Absolute quantification of the virion-sense (VS) and the complementary-sense (CS) strands was performed following the protocol described by^52^ with the following modifications: 15 ng of total DNA were used for the VS and CS strand synthesis step and 2 μl of a 1:2 dilution of the purified DNA, were used as the template for the qPCR. Total viral DNA was quantified by standard qPCR using 3 ng of total DNA.

### Libraries construction and sequencing

RNA-Seq libraries were generated and sequenced at CNAG (Centro Nacional de Análisis Genómico, Barcelona, Spain). Total RNA was assayed for quantity and quality using Qubit® RNA HS Assay (Life Technologies) and RNA 6000 Nano Assay on a Bioanalyzer 2100. The RNASeq libraries were prepared from total RNA using the TruSeq®Stranded mRNA LT Sample Prep Kit (Illumina Inc., Rev.E, October 2013). A total of 24 libraries were constructed (12 for each inoculation method; 3 biological replicates at 2, 7, 14 and 21 dpi). The libraries were sequenced on HiSeq2000 (Illumina, Inc) in paired-end mode with a read length of 76 bp using the TruSeq SBS Kit v4 (Table S2). Image analysis, base calling and quality scoring of the run were processed using the manufacturer’s software Real Time Analysis (RTA 1.18.64 or 1.18.66.3) and followed by generation of FASTQ sequence files by CASAVA.

sRNA libraries were generated and sequenced at CRG (Centre For Genomic Regulation, Barcelona, Spain). Libraries were prepared using the Illumina TruSeq small RNA sample prep kit according to the manufacturer’s instructions and were validated on an Agilent 2100 Bioanalyzer using a DNA High Sensitivity chip and quantified by qPCR using the Kapa Library Quantification kit for Illumina (Roche). Sequencing was performed on an Illumina HiSeq2500 using 50 bp single reads with HiSeq v4 sequencing chemistry. A total of 12 high-coverage libraries were generated and sequenced (6 for each inoculation method; 2 biological replicates at 7, 14 and 21 dpi) (Table S2).

Bisulfite sequencing (BS-Seq) library construction was performed at CNAG. Total genomic DNA (2 μg) was spiked with unmethylated bacteriophage λ DNA (5 ng of λ DNA per microgram of genomic DNA). The DNA was sheared to 50–500 bp in size using Covaris LE220 ultrasonicator, and fragments of 150–300 bp were size-selected using AMPure XP beads (Agencourt Bioscience). The libraries were constructed using the KAPA Library Preparation Kit with no PCR Library Amplification/Illumina series (Roche-Kapa Biosystems) together with the Illumina single index adaptors (Illumina). After adaptor ligation, the DNA was treated with sodium bisulfite using the EpiTect Bisulfite kit (Qiagen) following the manufacturer’s instructions. Enrichment for adaptor-ligated DNA was carried out through seven PCR cycles using KAPA HiFi HotStart Uracil + ReadyMix PCR 2x Kit (Roche-Kapa Biosystems). Four libraries were constructed (2 for each inoculation method; 2 biological replicates at 14 dpi) (Table S2). Library quality was monitored using the Agilent 2100 Bioanalyzer DNA 7500 assay, and the library concentration was estimated using quantitative PCR using the KAPA Library Quantification Kit for Illumina® Platforms, v1.14 (Roche-Kapa Biosystems). Paired-end DNA sequencing (2 × 101 bp) of the converted libraries was performed using the HiSeq2000 (Illumina) following the manufacturer’s protocol with HiSeq Control Software (HCS) 1.5.15.1 to achieve no less than 24x tomato genome coverage. Images analysis, base calling and quality scoring of the run were processed using the manufacturer’s software Real Time Analysis (RTA 1.13.48) and followed by generation of FASTQ sequence files.

### Accession numbers

Data discussed in this publication have been deposited in the NCBI Gene Expression Omnibus (GEO, http://www.ncbi.nlm.nih.gov/geo) and are accessible through the NCBI Short Read Archive accession number SRP164300.

### Bionformatics analysis

RNA-Seq paired-end reads were mapped against the TYLCV reference genome (Genbank Acc. No. AJ489258) using STAR version 2.5.1b^65^ with ENCODE parameters for long RNA. Viral transcriptional units were quantified using RSEM version 1.2.28^66^ with default parameters. To make sense/antisense coverage plots, we extracted alignments from both strands using coverageBed^67^ and plotting it using the GViz R package.

For sRNA data adaptor and ambiguous bases were first trimmed from the raw sRNA reads using a custom Perl script. Reads longer than 15 bp were then aligned to a combined RNA database consisting of tRNA (transfer RNA), snoRNA (small nucleolar RNA), snRNA (small nuclear RNA) and rRNA (ribosomal RNA), sequences of plants, and those could be aligned were discarded. The resulting cleaned reads were mapped to TYLCV genome, respectively, using Bowtie^68^, allowing one mismatch. The sRNA size distribution was summarized for both origins. The total number of reads, reads in forward and reverse orientation and reads starting with A, C, G and T for TYLCV sRNA size classes from 20 to 25 nt, were estimated using MISIS^69^. Plotting of vsRNAs along the viral genome was accomplished by Excel (Fig. 4) or MISIS (Figs S1 and S2).

From the BS-Seq data, potential PCR duplicates were removed: read pairs having identical bases at positions from 10 to 80 in both left and right reads were defined as duplicated pairs and then collapsed into unique read pairs. The resulting reads were further processed to remove adaptor and low-quality sequences using Trimmomatic^70^. The trimmed reads were then mapped to TYLCV genome genomes using a methylation-aware aligner Bismark v0.17.0 (–bowtie1 -n1)^71^. The methylation information was then extracted from the alignments by a script “bismark_methylation_extractor” provided in Bismark and the resulted cytosine reports were separated according to the cytosine context, CG, CHG and CHH. Plotting of the viral methylome was accomplished using ggplot2 (Fig. 5B) or Excel (Figs 6 and 7).

## Supplementary information

Suppl. Info

## References

[1] Hesketh EL (2018). The 3.3 Å structure of a plant geminivirus using cryo-EM. Nat Commun.

[2] Zerbini FM (2017). ICTV Virus Taxonomy Profile: Geminiviridae. Journal of General Virology.

[3] Jeske H, Lütgemeier M, Preiss W (2001). DNA forms indicate rolling circle and recombination-dependent replication of Abutilon mosaic virus. EMBO J.

[4] Preiss W, Jeske H (2003). Multitasking in replication is common among geminiviruses. J Virol.

[5] Jeske H (2009). Geminiviruses. Curr Top Microbiol Immunol.

[6] Díaz-Pendón JA (2010). Tomato yellow leaf curl viruses: ménage à trois between the virus complex, the plant and the whitefly vector. Mol Plant Pathol.

[7] Navas-Castillo J, Fiallo-Olivé E, Sánchez-Campos S (2011). Emerging virus diseases transmitted by whiteflies. Annu Rev Phytopathol.

[8] Cohen S, Nitzany FE (1960). A whitefly transmitted virus of cucurbits in Israel. Phytopathologia Mediterranea.

[9] Fondong VN (2013). Geminivirus protein structure and function. Mol Plant Pathol.

[10] Hanley-Bowdoin L, Settlage SB, Orozco BM, Nagar S, Robertson D (1999). Geminiviruses: models for plant DNA replication, transcription, and cell cycle regulation. Crit. Rev. Biochem. Mol. Biol..

[11] Laufs J (1995). *In vitro* cleavage and joining at the viral origin of replication by the replication initiator protein of tomato yellow leaf curl virus. Proc Natl Acad Sci USA.

[12] Fontes EP, Luckow VA, Hanley-Bowdoin L (1992). A geminivirus replication protein is a sequence-specific DNA binding protein. Plant Cell.

[13] Fontes EP, Gladfelter HJ, Schaffer RL, Petty IT, Hanley-Bowdoin L (1994). Geminivirus replication origins have a modular organization. Plant Cell.

[14] Argüello-Astorga GR, Guevara-González RG, Herrera-Estrella LR, Rivera-Bustamante RF (1994). Geminivirus replication origins have a group-specific organization of iterative elements: a model for replication. Virology.

[15] Borah BK, Zarreen F, Baruah G, Dasgupta I (2016). Insights into the control of geminiviral promoters. Virology.

[16] Pumplin N, Voinnet O (2013). RNA silencing suppression by plant pathogens: defence, counter-defence and counter-counter-defence. Nat Rev Micro.

[17] Yifhar T (2012). Failure of the tomato trans-acting short interfering RNA program to regulate AUXIN RESPONSE FACTOR3 and ARF4 underlies the wiry leaf syndrome. Plant Cell.

[18] Kravchik M, Damodharan S, Stav R, Arazi T (2014). Generation and characterization of a tomato DCL3-silencing mutant. Mol Plant Microbe Interact.

[19] Bologna NG, Voinnet O (2014). The diversity, biogenesis, and activities of endogenous silencing small RNAs in Arabidopsis. Annu Rev Plant Biol.

[20] Borges F, Martienssen RA (2015). The expanding world of small RNAsin plants. Nat Rev Mol Cell Biol.

[21] Law JA, Jacobsen SE (2010). Establishing, maintaining and modifying DNA methylation patterns in plants and animals. Nat Rev Genet.

[22] Matzke MA, Mosher RA (2014). RNA-directed DNA methylation: an epigenetic pathway of increasing complexity. Nat Rev Genet.

[23] Du J (2012). Dual binding of chromomethylase domains to H3K9me2-containing nucleosomes directs DNA methylation in plants. Cell.

[24] Stroud H, Greenberg MVC, Feng S, Bernatavichute YV, Jacobsen SE (2013). Comprehensive analysis of silencing mutants reveals complex regulation of the Arabidopsis methylome. Cell.

[25] Raja P, Wolf JN, Bisaro DM (2010). RNA silencing directed against geminiviruses: post-transcriptional and epigenetic components. Biochim. Biophys. Acta.

[26] Hanley-Bowdoin L, Bejarano ER, Robertson D, Mansoor S (2013). Geminiviruses: masters at redirecting and reprogramming plant processes. Nat Rev Micro.

[27] Pooggin M (2013). How can plant DNA viruses evade siRNA-directed DNA methylation and silencing?. IJMS.

[28] Ramesh, S. V., Sahu, P. P., Prasad, M., Praveen, S. & Pappu, H. R. Geminiviruses and plant hosts: a closer examination of the molecular arms race. *Viruses* **9** (2017). 10.3390/v9090256 PMC5618022 28914771

[29] Donaire L (2009). Deep-sequencing of plant viral small RNAs reveals effective and widespread targeting of viral genomes. Virology.

[30] Yang X (2011). Characterization of small interfering RNAs derived from the geminivirus/betasatellite complex using deep sequencing. PLoS One.

[31] Miozzi L, Pantaleo V, Burgyán J, Accotto GP, Noris E (2013). Analysis of small RNAs derived from Tomato yellow leaf curl Sardinia virus reveals a cross reaction between the major viral hotspot and the plant host genome. Virus Res.

[32] Xu C (2017). Diversity, distribution, and evolution of tomato viruses in China uncovered by small RNA sequencing. J Virol.

[33] Aregger M (2012). Primary and Secondary siRNAs in Geminivirus-induced gene silencing. PLoS Pathog.

[34] Rogans SJ, Allie F, Tirant JE, Rey MEC (2016). Small RNA and methylation responses in susceptible and tolerant landraces of cassava infected with South African cassava mosaic virus. Virus Res.

[35] Pilartz M, Jeske H (2003). Mapping of abutilon mosaic geminivirus minichromosomes. J Virol.

[36] Pilartz M, Jeske H (1992). Abutilon mosaic geminivirus double-stranded DNA is packed into minichromosomes. Virology.

[37] Raja P, Sanville BC, Buchmann RC, Bisaro DM (2008). Viral genome methylation as an epigenetic defense against geminiviruses. J Virol.

[38] Castillo-González, C. *et al*. Geminivirus-encoded TrAP suppressor inhibits the histone methyltransferase SUVH4/KYP to counter host defense. *Elife* 1–31, 10.7554/eLife.06671.001 (2015). 10.7554/eLife.06671 PMC4606454 26344546

[39] Ceniceros-Ojeda EA, Rodríguez-Negrete EA, Rivera-Bustamante RF (2016). Two populations of viral minichromosomes are present in a geminivirus-infected plant showing symptom remission (recovery). J Virol.

[40] Bian X-Y, Rasheed MS, Seemanpillai MJ, Ali Rezaian M (2006). Analysis of silencing escape of tomato leaf curl virus: an evaluation of the role of DNA methylation. MPMI.

[41] Rodriguez-Negrete EA, Carrillo-Tripp J, Rivera-Bustamante RF (2009). RNA Silencing against geminivirus: complementary action of posttranscriptional gene silencing and transcriptional gene silencing in host recovery. J Virol.

[42] Yadav RK, Chattopadhyay D (2011). Enhanced viral intergenic region-specific short interfering RNA accumulation and DNA methylation correlates with resistance against a geminivirus. MPMI.

[43] Yang X (2011). Suppression of methylation-mediated transcriptional gene silencing by βC1-SAHH protein interaction during geminivirus-betasatellite infection. PLoS Pathog.

[44] Zhang Z (2011). BSCTV C2 attenuates the degradation of SAMDC1 to suppress DNA methylation-mediated gene silencing in Arabidopsis. Plant Cell.

[45] Paprotka T, Deuschle K, Metzler V, Jeske H (2011). Conformation-selective methylation of geminivirus DNA. J Virol.

[46] Sahu PP, Sharma N, Puranik S, Prasad M (2014). Post-transcriptional and epigenetic arms of RNA silencing: a defense machinery of naturally tolerant tomato plant against Tomato leaf curl New Delhi virus. Plant Mol Biol Rep.

[47] Jackel JN, Storer JM, Coursey T, Bisaro DM (2016). Arabidopsis RNA polymerases IV and V are required to establish H3K9 methylation, but not cytosine methylation, on geminivirus chromatin. J Virol.

[48] Deuschle K, Kepp G, Jeske H (2016). Differential methylation of the circular DNA in geminiviral minichromosomes. Virology.

[49] Torchetti, E. M. *et al*. A nuclear-replicating viroidantagonizes infectivity andaccumulation of a geminivirusby upregulating methylation-related genes and inducing hypermethylation of viralDNA. *Sci. Rep*. 1–16, 10.1038/srep35101 (2016). 10.1038/srep35101 PMC5064398 27739453

[50] Raja P, Jackel JN, Li S, Heard IM, Bisaro DM (2014). Arabidopsis double-stranded RNA binding protein DRB3 participates in methylation-mediated defense against geminiviruses. J Virol.

[51] Ghoshal B, Sanfaçon H (2015). Symptom recovery in virus-infected plants: revisiting the role of RNA silencing mechanisms. Virology.

[52] Rodríguez-Negrete EA (2014). A sensitive method for the quantification of virion-sense and complementary-sense DNA strands of circular single-stranded DNAviruses. Sci. Rep..

[53] Mi S (2008). Sorting of small RNAs into Arabidopsis Argonaute complexes is directed by the 5′ terminal nucleotide. Cell.

[54] Kim VN (2008). Sorting out small RNAs. Cell.

[55] Mallory A, Vaucheret H (2010). Form, function, and regulation of Argonaute proteins. Plant Cell.

[56] Shivaprasad PV (2005). Promoters, transcripts, and regulatory proteins of Mungbean yellow mosaic geminivirus. J Virol.

[57] Tu J, Sunter G (2007). A conserved binding site within the Tomato golden mosaic virus AL-1629 promoter is necessary for expression of viral genes important for pathogenesis. Virology.

[58] Elmer JS (1988). Agrobacterium-mediated inoculation of plants with tomato golden mosaic virus DNAs. Plant Molecular Biology.

[59] Noris E (2004). Tomato yellow leaf curl Sardinia virus can overcome transgene-mediated RNA silencing of two essential viral genes. Journal of General Virology.

[60] Chellappan P, Vanitharani R, Pita J, Fauquet CM (2004). Short interfering RNA accumulation correlates with host recovery in DNA virus-infected hosts, and gene silencing targets specific viral sequences. J Virol.

[61] Zemach A (2013). The Arabidopsis nucleosome remodeler DDM1 allows DNA methyltransferases to access H1-containing heterochromatin. Cell.

[62] Stroud H (2014). Non-CG methylation patterns shape the epigenetic landscape in Arabidopsis. Nat Struct Mol Biol.

[63] Morilla G (2005). Pepper (Capsicum annuum) Is a dead-end host for Tomato yellow leaf curl virus. Phytopathology.

[64] Pereira-Carvalho R (2015). Recessive resistance derived from Tomato cv. Tyking-limits drastically the spread of Tomato yellow leaf curl virus. Viruses.

[65] Dobin A (2013). STAR: ultrafast universal RNA-seq aligner. Bioinformatics.

[66] Li B, Dewey CN (2011). RSEM: accurate transcript quantification from RNA-Seq data with or without a reference genome. BMC Bioinformatics.

[67] Quinlan AR, Hall IM (2010). BEDTools: a flexible suite of utilities for comparing genomic features. Bioinformatics.

[68] Langmead, B., Trapnell, C., Pop, M. & Salzberg, S. L. Ultrafast and memory-efficient alignment of short DNA sequences to the human genome. *Software* 1–10, 10.1186/gb-2009-10-3-r25 (2009). 10.1186/gb-2009-10-3-r25 PMC2690996 19261174

[69] Seguin J, Otten P, Baerlocher L, Farinelli L, Pooggin MM (2016). MISIS-2: A bioinformatics tool for in-depth analysis of small RNAs and representation of consensus master genome in viral quasispecies. Journal of Virological Methods.

[70] Bolger, A. M., Lohse, M. & Usadel, B. Trimmomatic: a flexible trimmer for Illumina sequence data. *Bioinformatics* 1–7, 10.1093/bioinformatics/btu170/-/DC1 (2014). 10.1093/bioinformatics/btu170 PMC4103590 24695404

[71] Krueger F, Andrews SR (2011). Bismark: a flexible aligner and methylation caller for Bisulfite-Seq applications. Bioinformatics.

