# Supplementary material for: Integrated single-base resolution maps of transcriptome, sRNAome and methylome of Tomato yellow leaf curl virus (TYLCV) in tomato
Source: Sci Rep. 2019 Feb 27;9:2863. doi: 10.1038/s41598-019-39239-6 (PMC6393547; doi:10.1038/s41598-019-39239-6)
Supplement: Supplementary file 1 — Suppl. Info [file 41598_2019_39239_MOESM1_ESM.pdf]

Integrated single-base resolution maps of transcriptome, sRNAome and methylome of *Tomato yellow leaf curl virus* (TYLCV) in tomato.

Álvaro Piedra-Aguilera[1δ], Chen Jiao[3δ], Ana P. Luna[1], Francisco Villanueva[2], Marc Dabad[4], Anna Esteve-Codina[4,5] , Juan A. Díaz-Pendón[2], Zhangjun Fei[3], Eduardo R. Bejarano[1] and Araceli G. Castillo[1]*.

[1] Instituto de Hortofruticultura Subtropical y Mediterránea La Mayora (IHSM-UMA-CSIC). Área de Genética, Facultad de Ciencias, Universidad de Málaga, E-29071 Málaga, Spain.

[2] Instituto de Hortofruticultura Subtropical y Mediterránea La Mayora (IHSM-UMA-CSIC). Plant Virology group, E. E. La Mayora CSIC, Algarrobo-Costa, E-29750 Málaga, Spain.

[3] Boyce Thompson Institute for Plant Research, Cornell University, Ithaca, New York, USA.

[4] CNAG-CRG, Barcelona Institute of Science and Technology (BIST), E-08028, Barcelona, Spain.

[5] Universitat Pompeu Fabra (UPF), E-08003, Barcelona, Spain.

[δ] Authors contributed equally to this work.

*For correspondence (Araceli G Castillo. Instituto de Hortofruticultura Subtropical y Mediterránea "La Mayora" (IHSM-UMA-CSIC), Área de Genética, Facultad de Ciencias, Universidad de Málaga, Campus de Teatinos s/n, E-29071 Málaga, Spain. Tel: +34 952131677; e-mail: ara@uma.es.

	Agrob-TYLCV				*Bemisia-TYLCV*			
**Viral DNA**	**2 dpi**	**7 dpi**	**14 dpi**	**21dpi**	**2 dpi**	**7 dpi**	**14 dpi**	**21dpi**
VS (x10^6)	ndt	16±5.5	140±43	97±3.0	ndt	3.5±2.0	100±32	183±56
CS (x10^6)	ndt	1.1±0.4	6.9±1.5	4.3±0.7	ndt	0.22±0.1	6.9±0.9	9.0±0.5
VS/CS ratio	ndt	13.6	19.9	22.6	ndt	16.9	14.6	20.2

Table S1. Absolute quantification of virion-sense (VS) or complementary-sense (CS) strands of TYLCV during the infection. Tomato plants were infected by either agroinoculation (*Agrob-TYLCV*) or by *B. tabaci* (*Bemisia-TYLCV*). Data from different time points during the systemic infection are shown (2, 7, 14 and 21 dpi). Values represent the average ± SE of viral molecules (x10^6) per ng of DNA from the three biological replicates. The ratio VS/CS at each dpi, is indicated for each dpi. Ndt: non-detected

	RNA-Seq: Raw read pairs (2x75-nt)				***sRNA-Seq: Raw reads 16-50 nt (1x50-nt)***			***BS-Seq: Raw read pairs***
	2 dpi	***7 dpi***	***14dpi***	***21 dpi***	***7 dpi***	***14dpi***	***21 dpi***	***14dpi***
Agrob-TYLCV_R1	28,308,689	34,325,652	31,509,779*	33,716,991	77,628,196	74,999,009^	75,720,534	137,255,859
Agrob-TYLCV_R2	32,494,007	36,180,219*	41,152,086	34,535,147*	82,137,526^	66,201,477	70,746,164^	129,137,303
Agrob-TYLCV_R3	32,770,672	27,175,266	30,003,595	30,713,610	nd	nd	nd	nd
Bemisia-TYLCV_R1	29,322,004	29,528,784	36,255,934*	25,898,937*	75,012,927	83,630,170^	79,764,671^	144,368,733
Bemisia-TYLCV_R2	29,357,804	36,254,389*	30,167,165	27,239,950	77,704,976^	81,084,958	82,169,778	128,106,473
Bemisia-TYLCV_R3	46,535,861	36,759,995	30,534,930	31,000,225	nd	nd	nd	nd

* Data obtained from this biological replicate is plotted on Figure 2
^ Data obtained from this biological replicate is plotted on Figure 4
nd: non-determined

Table S2. Raw reads for TYLCV-tomato infected samples from RNA-Seq, sRNA-Seq and BS-Seq analyses. Tomato plants were infected by either agroinoculation (*Agrob-TYLCV*) or using the whitefly *B. tabaci* (*Bemisia-TYLCV*) and data from different time points during the systemic infection (2, 7, 14 and 21 dpi) and from the different biological replicates (R1, R2 and R3) are shown.

	Reads mapped to TYLCV (No.)				Reads mapped to TYLCV (%)			
Agrob-TYLCV	***2 dpi***	***7 dpi***	***14dpi***	***21 dpi***	***2 dpi***	***7 dpi***	***14dpi***	***21 dpi***
R1	0	14477	86841	92538	0.000	0.022	0.110	0.141
R2	34	38532	57658	85343	0.000	0.056	0.101	0.132
R3	0	17785	61593	77002	0.000	0.035	0.103	0.130
Average					*0.00±0.000*	*0.04±0.017*	*0.10±0.005*	*0.13±0.006*
Bemisia-TYLCV	***2 dpi***	***7 dpi***	***14dpi***	***21 dpi***	***2 dpi***	***7 dpi***	***14dpi***	***21 dpi***
R1	3	5043	115250	95968	0.000	0.009	0.164	0.193
R2	10	26221	116090	91716	0.000	0.037	0.199	0.176
R3	10	6350	89807	106900	0.000	0.009	0.152	0.179
Average					*0.00±0.000*	*0.02±0.016*	*0.17±0.025*	*0.18±0.009*

Table S3. TYLCV transcripts reads from tomato plants infected by either agroinoculation (*Agrob-TYLCV*) or by *B. tabaci* (*Bemisia-TYLCV*). Data from each biological replicate (R1, R2 and R3) at different time points during the systemic infection are shown (2, 7, 14 and 21 dpi). Values correspond to the total number of reads that mapped to TYLCV genome (No.) or the percentage (%) that they represent relative to the total transcripts reads. The average ± SE from the three biological replicates is indicated for the percentage of TYLCV-mapped transcripts.

		Percentage (%) of each size-class of TYLCV vsRNA						
		**20-nt**	**21-nt**	**22-nt**	**23-nt**	**24-nt**	**25-nt**	**20-25 nt**
Agrob-TYLCV								
R1	*7 dpi*	0.01	0.10	0.05	0.01	0.02	0.00	0.20
R2	*7 dpi*	0.06	0.35	0.26	0.04	0.06	0.01	0.78
R1	*14 dpi*	0.40	2.11	1.50	0.25	0.41	0.05	4.72
R2	*14 dpi*	0.43	2.17	1.52	0.25	0.42	0.06	4.83
R1	*21 dpi*	0.52	2.38	1.70	0.32	0.43	0.07	5.40
R2	*21 dpi*	0.54	2.57	1.62	0.30	0.47	0.06	5.57
Bemisia-TYLCV								
R1	*7 dpi*	0.00	0.01	0.01	0.00	0.00	0.00	0.02
R2	*7 dpi*	0.01	0.08	0.06	0.01	0.01	0.00	0.17
R1	*14 dpi*	0.29	1.64	1.03	0.17	0.26	0.04	3.42
R2	*14 dpi*	0.24	1.38	0.93	0.15	0.23	0.04	2.96
R1	*21 dpi*	0.51	2.53	1.31	0.26	0.38	0.05	5.04
R2	*21 dpi*	0.54	2.63	1.41	0.28	0.37	0.06	5.28

Table S4. Size distribution of 20-25 nt TYLCV vsRNAs. Tomato plants were infected by either agroinoculation (*Agrob-TYLCV*) or using *B. tabaci* (*Bemisia-TYLCV*) and data from each biological replicate (R1 and R2) at different time points during the systemic infection are shown (7, 14 and 21 dpi). Values show the percentage (%) that each size-class of TYLCV vsRNA represent relativized to the total sRNA reads (19-35 nt).

	Reduntant vsRNA (%)					
	**20-nt**	**21-nt**	**22-nt**	**23-nt**	**24-nt**	**25-nt**
Agrob-TYLCV						
7 dpi	4.9	48.5	33.6	4.3	7.5	1.2
14 dpi	5.6	46.7	33.0	4.9	8.8	1.0
21 dpi	5.6	47.4	32.1	5.4	8.4	1.2
Bemisia-TYLCV						
7 dpi	4.9	49.4	32.9	4.5	6.9	1.4
14 dpi	5.5	49.6	31.8	4.5	7.5	1.1
21 dpi	6.3	53.0	27.7	4.8	7.2	1.1

	Unique vsRNA (%)					
	**20-nt**	**21-nt**	**22-nt**	**23-nt**	**24-nt**	**25-nt**
Agrob-TYLCV						
7 dpi	13.5	25.9	24.8	13.2	16.4	6.2
14 dpi	16.4	20.0	19.8	16.1	17.5	10.3
21 dpi	16.0	20.1	20.0	16.4	17.7	9.8
Bemisia-TYLCV						
7 dpi	10.8	32.2	28.3	10.8	13.7	4.1
14 dpi	16.4	20.4	20.2	15.9	17.2	9.9
21 dpi	16.6	20.0	19.5	16.2	17.2	10.4

Table S5. Percentage of redundant and unique 20-25 nt TYLCV vsRNAs from tomato plants infected by either agroinoculation (*Agrob-TYLCV*) or by *B. tabaci* (*Bemisia-TYLCV*). Values show the percentage (%) that each size-class of TYLCV vsRNA represent relativized to the total vsRNA reads (20-25 nt). The average from two independent biological replicates at different time points during the systemic infection (7, 14 and 21 dpi) are shown.

Redundant vsRNA

	**20-nt**		**21-nt**		**22-nt**		**23-nt**		**24-nt**		**25-nt**		**20-25-nt**	
	**No.**	**% VS**	**No.**	**% VS**	**No.**	**% VS**	**No.**	**% VS**	**No.**	**% VS**	**No.**	**% VS**	**No.**	**% VS**
Agrob-TYLCV														
7 dpi	12302	**50.7**	122459	**47.8**	84956	**51.1**	10866	**38.5**	19037	**39.6**	2931	**25.1**	252551	**47.8**
14 dpi	119774	**51.9**	1007651	**43.2**	711545	**48.7**	104932	**37.8**	190008	**40.3**	22520	**29.9**	2156433	**44.8**
21 dpi	132945	**51.3**	1123872	**41.9**	760109	**47.2**	127847	**36.4**	199502	**40.2**	27478	**26.8**	2371754	**43.5**
Bemisia-TYLCV														
7 dpi	1738	**50.9**	17451	**49.1**	11600	**51.7**	1578	**36.6**	2438	**34.2**	494	**22.3**	35300	**48.1**
14 dpi	89013	**50.1**	797537	**45.8**	510951	**50.3**	72861	**38.9**	120487	**39.6**	17853	**29.7**	1608700	**46.5**
21 dpi	161058	**52.5**	1355323	**42.8**	709965	**49.1**	123573	**38.1**	182897	**40.1**	26859	**28.7**	2559674	**44.6**

Unique vsRNA

	**20-nt**		**21-nt**		**22-nt**		**23-nt**		**24-nt**		**25-nt**		**20-25-nt**	
	**No.**	**% VS**	**No.**	**% VS**	**No.**	**% VS**	**No.**	**% VS**	**No.**	**% VS**	**No.**	**% VS**	**No.**	**% VS**
Agrob-TYLCV														
7 dpi	1764	**49.3**	3385	**50.0**	3241	**50.0**	1719	**45.8**	2138	**46.2**	804	**39.8**	13050	**48.1**
14 dpi	3829	**50.2**	4684	**49.7**	4642	**49.9**	3763	**48.6**	4105	**48.9**	2401.5	**45.3**	23424	**49.0**
21 dpi	3653	**49.7**	4567	**49.4**	4553	**49.5**	3736	**48.8**	4024	**48.9**	2235.5	**46.2**	22768	**49.0**
Bemisia-TYLCV														
7 dpi	532	**48.6**	1582	**49.7**	1393	**50.6**	530	**58.5**	673	**58.3**	201	**64.8**	4911	**47.1**
14 dpi	3415	**49.8**	4253	**50.3**	4194	**50.3**	3316	**51.3**	3580	**51.1**	2056	**54.3**	20813	**49.0**
21 dpi	3626	**50.3**	4360	**50.5**	4263	**50.4**	3540	**51.4**	3756	**51.1**	2269.5	**53.1**	21813	**49.1**

Table S6. Total number (No.) and percentage mapped to the virion-sense strand (% VS) of TYLCV 20-25 nt vsRNA. Tomato plants were infected by either agroinoculation (*Agrob-TYLCV*) or using *B. tabaci* (*Bemisia-TYLCV*) and the average value from two independent biological replicates at different time points the during systemic infection (7, 14 and 21 dpi) are shown.

	Uniquely mapped read pairs	*Average coverage TYLCV*	*Average coverage TYLCV-mean*
Agrob-TYLCV_R1	78932	5588	5096
Agrob-TYLCV_R2	64817	4604	
Bemisia-TYLCV_R1	107547	7626	7585
Bemisia-TYLCV_R2	106295	7544	

Table S7. Average methylome coverage of TYLCV genome at 14 dpi. Tomato plants were infected by either agroinoculation (*Agrob-TYLCV*) or using the whitefly *B. tabaci* (*Bemisia-TYLCV*) and data from the two biological replicates (R1 and R2) are shown.

	Experimental data			Estimated data	
	**Total meC**	**Total unmeC**	**Methylation (%)**	**meC-dsDNA**	**Methyl.-dsDNA (%)**
Agrob-TYLCV_R1<br>(14.1% strands in dsDNA)					
CG	5,147	467,270	1.09	66,611	7.7
CHG	11,485	517,289	2.17	74,557	15.4
CHH	56,788	1,830,324	3.01	266,083	21.3
Total	*73,420*	*2,814,883*	*2.54*	*407,251*	***18.0***
Agrob-TYLCV_R2<br>(8.6% strands in dsDNA)					
CG	3,895	385,954	1.00	33,527	11.6
CHG	8,992	426,385	2.07	37,442	24.0
CHH	42,768	1,487,033	2.80	131,563	32.5
Total	*55,655*	*2,299,372*	*2.36*	*202,532*	***27.5***
Bemisia-TYLCV_R1<br>(13.5% strands in dsDNA)					
CG	3,286	638,877	0.51	86,692	3.8
CHG	7,383	723,674	1.01	98,693	7.5
CHH	38,251	2,506,805	1.50	343,583	11.1
Total	*48,920*	*3,869,356*	*1.25*	*528,967*	***9.2***
Bemisia-TYLCV_R2<br>(22.5% strands in dsDNA)					
CG	2,671	642,910	0.41	145,256	1.8
CHG	6,037	728,050	0.82	165,170	3.7
CHH	30,588	2,509,445	1.20	571,507	5.4
Total	*39,296*	*3,880,405*	*1.00*	*881,933*	***4.5***

Table S8. Total number of methylated (meC) and unmethylated cytosines (unmeC) and the percentage of methylation (%) of TYLCV genome at 14 dpi obtained from tomato plants infected by agroinoculation (*Agrob-TYLCV*) or using *B. tabaci* (*Bemisia-TYLCV*). The data from the two biological replicates (R1 and R2) are shown (Experimental data). The number of methylated cytosines present in dsDNA strands (meC-dsDNA) shown in “Estimated data” was calculated according to the percentage of strands in dsDNA form from each biological replicate (% underneath each replica name, with a mean value of 11% for TYLCV-agroinfiltrated plants and 18% for *Bemisia*-treated plants). These percentages were obtained from the quantification of CS and VS strands (Figure 1), considering that all CS strands form dsRNA (Rodriguez-Negrete et al., 2014). The estimated percentage of methylation (%) of dsDNA is also indicated.

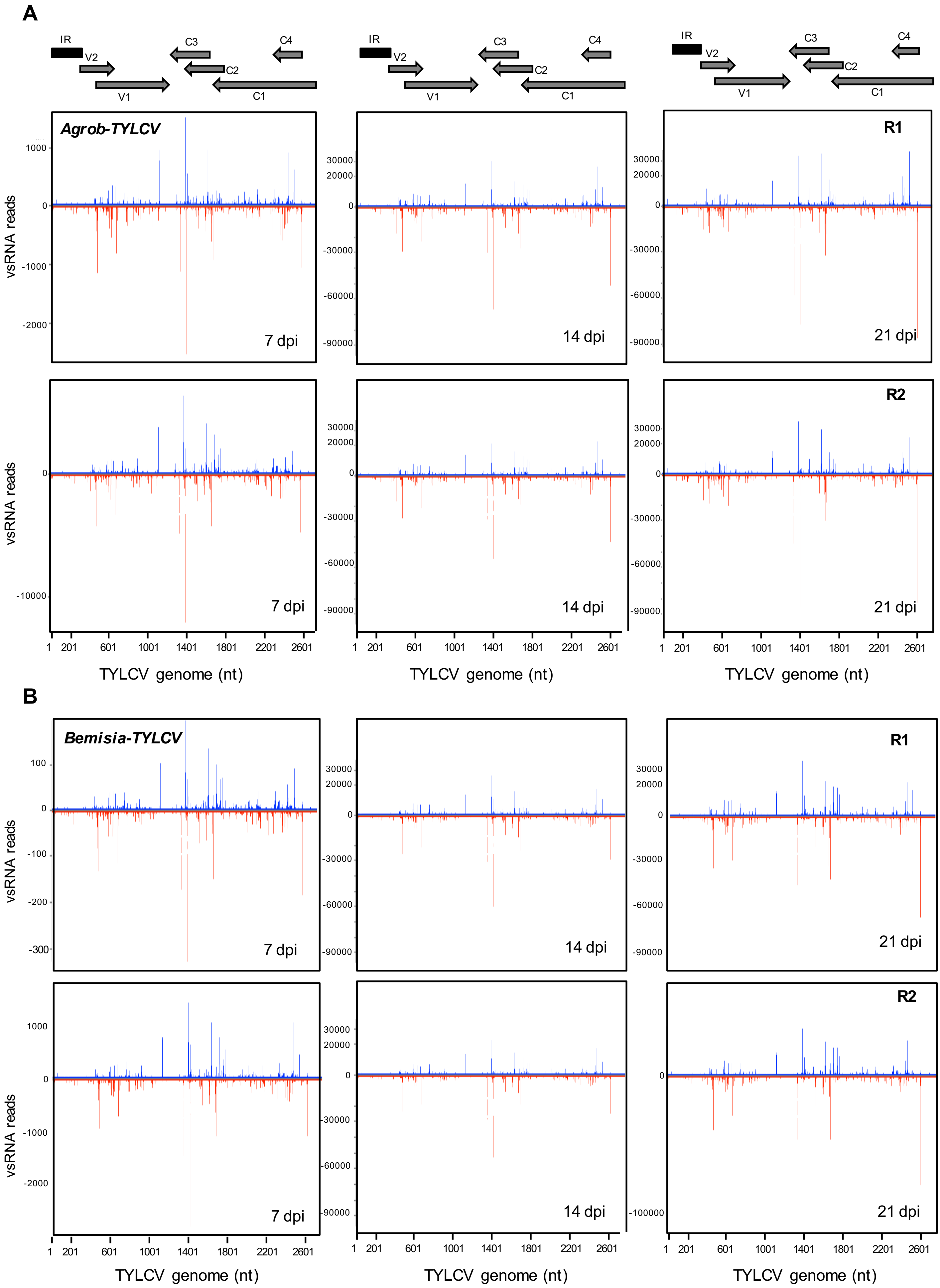
A
IR
V2
V1
C3
C2
C4
C1
Agrob-TYLCV
R1
R2
vsRNA reads
7 dpi
14 dpi
21 dpi
TYLCV genome (nt)
B
Bemisia-TYLCV
1
201
601
1001
1401
1801
2201
2601

Figure S1

Figure S1. Maps of vsRNA from TYLCV-infected tomato plants by either **(A)** agroinoculation (*Agrob-TYLCV*) or using **(B)** the whitefly *B. tabaci* (*Bemisia-TYLCV*). Data from both biological replicates for each condition (R1 and R2) at different time points during the infection (7, 14 and 21 dpi) are shown. The graphs plot the number of 20-25 nt vsRNA at each nucleotide position of TYLCV genome (2781 nt). Bars above the axis (blue) represent sense reads starting at each respective position and those below (red) represent antisense reads ending at that position. The genome organization of TYLCV is shown schematically above the graphs and the rightward ORFs (C1, C4, C2 and C3), the leftward ORFs (V1 and V2) and the intergenic region (IR) are indicated. The predicted ORFs are shown as grey arrows and the IR as a dark rectangle.

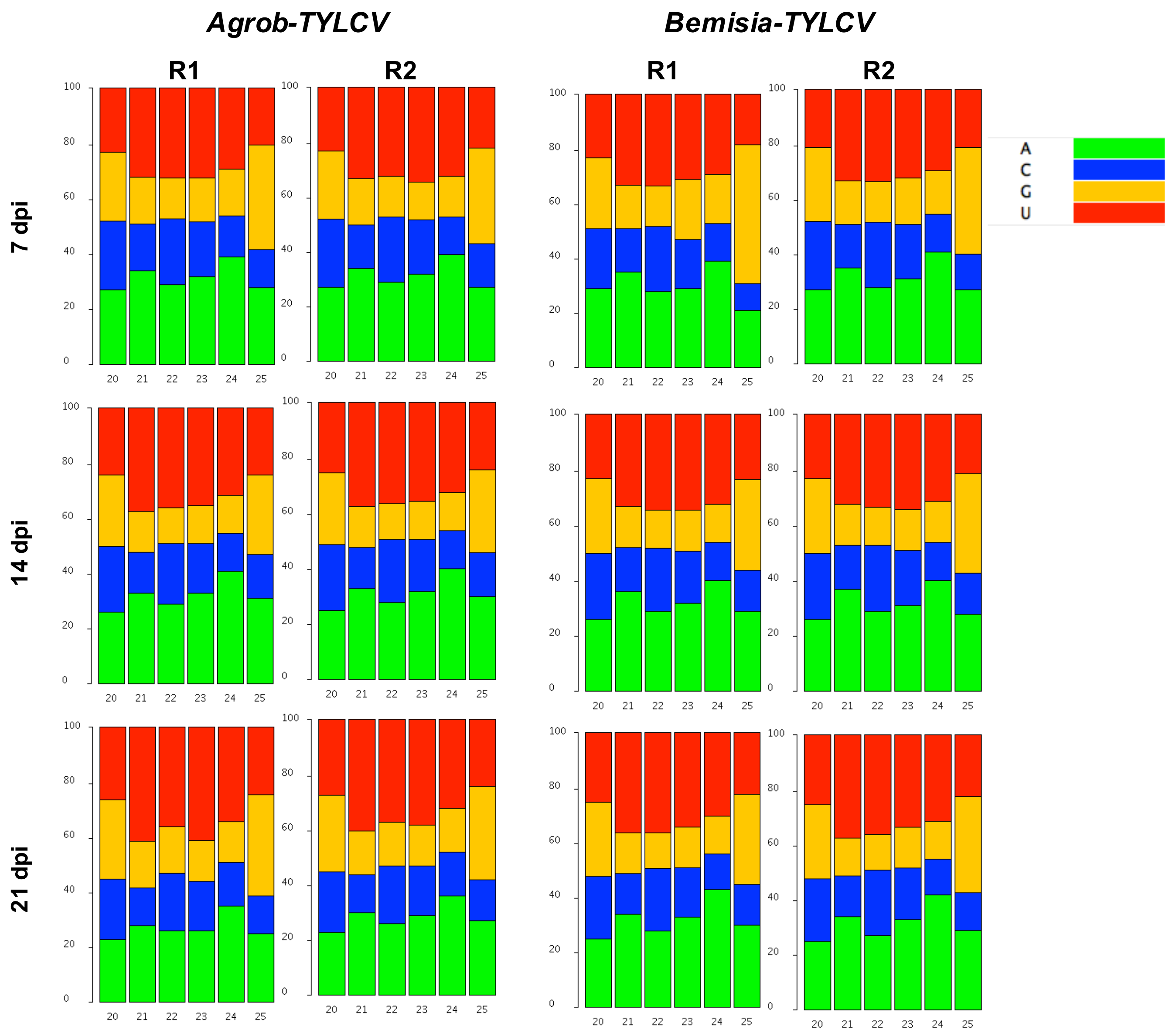

Figure S2. Abundance of vsRNAs 5′-terminal nucleotide with respect to the amount of individual nucleotides in the TYLCV genome. Tomato plants were infected by either agroinoculation (*Agrob-TYLCV*) or using the whitefly *B. tabaci* (*Bemisia-TYLCV*) and both biological replicates for each condition (R1 and R2) at different time points during the infection (7, 14 and 21 dpi) are shown. The Y-axis represents the percentage (%) of each nucleotide with respect to the total amount of that nucleotide.
